# Interoceptive predictors of daily functioning in aging and their interaction with exteroceptive bodily representations

**DOI:** 10.3389/fpsyg.2026.1689759

**Published:** 2026-02-06

**Authors:** Maria Rosaria Pasciucco, Francesco Bubbico, Scila Nunziata, Sabrina Iuliano, Gennaro Ruggiero, Mauro Gianni Perrucci, Pierpaolo Croce, Marcello Costantini, Francesca Ferri

**Affiliations:** 1 Department of Neuroscience, Imaging and Clinical Sciences, "G. d'Annunzio" University of Chieti and Pescara, Chieti, Italy; 2 Laboratory of Cognitive Science and Immersive Virtual Reality, CS-IVR, Department of Psychology, University of Campania L. Vanvitelli, Viale Ellittico, Caserta, Italy; 3 Institute for Advanced Biomedical Technologies, "G. d'Annunzio" University, Chieti, Italy; 4 UdA-TechLab, Research Center, University “G. d’Annunzio” of Chieti-Pescara, Chieti, Italy; 5 Department of Psychology, "G. d'Annunzio" University of Chieti and Pescara, Chieti, Italy

**Keywords:** assessment battery, body representation, healthy aging, interoception, multisensory processing

## Abstract

Aging leads to progressive changes in bodily functions that affect cognition and everyday life. Older adults often experience difficulties in daily functioning due to declines in two interconnected domains: interoception, the ability to sense and regulate internal bodily states, and exteroceptive body representations. While previous studies have noted these connections, systematic investigation into how interoception and exteroceptive body representations jointly influence daily functioning remains limited. This study had two main aims: (1) to develop a battery of tests assessing interoceptive states, multisensory perception, and exteroceptive body representations; and (2) to examine whether early interoceptive changes predict functional decline, potentially interacting with multisensory perception and bodily representation processes. We tested 60 healthy adults (aged 21–87) using a multimodal battery measuring interoceptive accuracy, sensibility, and awareness, along with assessments of body image, peripersonal space, tactile acuity, and sensorimotor functions. Daily functioning was evaluated using the SF-36 Health Survey. A Partial Least Squares Regression approach identified predictors of functional outcomes. Interoceptive sensibility, particularly self-regulation and body trust, was a key predictor of limitations in physical health, emotional wellbeing, and fatigue. Mean heart rate also contributed to fatigue perception. Exteroceptive body representations, including body image and peripersonal space processing, interacted with interoception in predicting daily functioning. These findings highlight the link between internal and external body processing in aging. Identifying these predictors not only guides the refinement of the test battery but also informs the development of targeted assessments and interventions aimed at promoting autonomy and enhancing quality of life in older adults.

## 1 Introduction

According to the World Health Organization (2002), the number of people aged 80 and over is expected to triple by 2050, posing significant challenges for healthcare systems and social infrastructures (International Monetary Fund, 2012). In this context, promoting healthy and active aging has become a global priority, requiring a deeper understanding of the factors that support physical autonomy, emotional wellbeing, and quality of life in older age. Active aging is a process that involves becoming aware of one’s own resources, which is essential to optimize opportunities for health, social participation, and high quality of life as people age (Stenner et al., 2011).

A crucial aspect in this process is bodily awareness, as it plays a fundamental role in adapting to age-related changes (Kuehn et al., 2018; Raimo et al., 2021). In aging, individuals experience a gradual process of changes due to concurrent decline of physical and cognitive functions. Concurrently, older adults face increasing difficulties in performing activities of daily living that range from basic activities, such as bathing, getting dressed or eating, to more complex, or instrumental activities, such as using public transportation or shopping (Jekel et al., 2015). This loss of daily functioning can significantly impact the independence of the elderly population, which, in turn, leads to negative consequences on their wellbeing (Covinsky et al., 2003; Stuck et al., 1999) and mental health (Wang et al., 2024). The rapid pace of population aging (Ageing and Health, 2025) highlights the urgent need to understand the mechanisms behind the decline in daily functioning and to develop preventive strategies to mitigate its impact.

Beyond traditional medical perspectives, aging research increasingly adopts a frailty framework, which conceptualizes vulnerability in later life as a multidimensional biopsychosocial (non-medical) syndrome (Cohen et al., 2023). Frailty encompasses cognitive, sensory, physical, and social domains such as slowness, weakness, exhaustion, polypharmacy, unintentional weight loss due to sarcopenia, and malnutrition, depression, dementia and affects a large portion of adults over 50 years old, with about 17% classified as “frail” and 45% as “pre-frail” (Cohen et al., 2023). Depending on the degree of impairment, individuals can be categorized as fit (non-frail), pre-frail, frail, or end-stage frail. Several models conceptualize frailty as a continuum across multiple domains. For instance, van Oostrom et al. (2017) proposed a four-domain model including physical, psychological, cognitive, and social frailty. These domains closely align with the interoceptive and exteroceptive processes investigated in the present study, which together may capture early, subtle changes that precede overt functional decline. Integrating this perspective allows us to frame interoceptive and multisensory bodily processes as potential embodied markers of vulnerability or resilience within the broader frailty continuum.

The difficulties in performing daily activities arise from the progressive loss of integrity of at least two intertwined dimensions: the interoceptive dimension (Ulus and Aisenberg-Shafran, 2022), which governs the ability to sense and regulate internal bodily states (Schmitt and Schoen, 2022), and the exteroceptive dimension, encompassing multisensory perception and body representations (Liu et al., 2025; Vlachou et al., 2025). These two domains jointly contribute to what can be described as functional body capacity, namely the individual’s ability to maintain efficient bodily function and sensorimotor adaptability necessary for the execution of everyday activities (Guralnik et al., 1996). Tactile perception and sensorimotor abilities, therefore, should be regarded as core components of the exteroceptive dimension, as they reflect how the body perceives and interacts with the external world (Mueller and Grunwald, 2023). Tactile processing provides essential information for distinguishing relevant environmental cues, supporting functions such as spatial discrimination, object identification, and the integration of contact-based signals into coherent representations of the peripersonal space. Sensorimotor abilities, in parallel, rely on the continuous coupling between sensory feedback and motor output, allowing the individual to maintain coordinated movement, adjust actions to environmental constraints, and update body representations during active exploration (Shadmehr and Krakauer, 2008; Wolpert et al., 2011). Together, these processes contribute to an effective exteroceptive system that supports adaptive behaviour and functional engagement with the surrounding environment.

Research shows that the progressive changes in bodily functions during aging lead to cognitive decline (Murman, 2015) and reduction in daily life functioning (Kuehn et al., 2018). These physiological and neurological changes are closely linked to the deterioration in interoceptive abilities, such as the accuracy and sensibility of perceiving internal bodily signals (Khalsa et al., 2009). As a result, older adults may experience difficulties in tasks requiring bodily awareness, emotional regulation, and interaction with their environment (Charles and Carstensen, 2010; Sardella et al., 2023). These difficulties often manifest as a decline in functional body capacity, an important indicator of health.

Functional body capacity refers to the integrated set of physical, sensory, and cognitive resources that enable individuals to effectively regulate their actions and interactions with the environment in daily life (Kekäläinen et al., 2023; Wróblewska et al., 2023). This construct is consistent with current models of functional capacity and intrinsic capacity proposed in aging research, which conceptualize body-related functions as multidimensional and dynamic determinants of independence and wellbeing (Beard et al., 2015; Cesari et al., 2018; Laughter et al., 2020; World Health Organization, 2020). Within this framework, functional body capacity reflects not only physical abilities (e.g., strength, balance, mobility) but also body-related perceptual and interoceptive processes that support adaptive self-regulation and environmental interaction (Schmitt and Schoen, 2022). Reduced functional body capacity not only diminishes the quality of life but also leads to a greater reliance on medical care and support services (Guralnik et al., 1996).

Interoception essentially refers to the perception of sensations stemming from inside the body such as heartbeats, breaths, temperature, hunger, and thirst (Craig, 2009; Critchley et al., 2004; Mayer et al., 2006), and it is a relevant aspect for better understanding the mechanisms underlying active aging, particularly in relation to physical functioning, wellbeing and daily functioning. Interoception can be divided into three distinct dimensions (Garfinkel et al., 2015): interoceptive accuracy, the objective ability to detect internal bodily sensations; interoceptive sensibility, the self-evaluation or confidence in recognizing these internal bodily sensations; interoceptive awareness, the metacognitive awareness of one’s interoceptive abilities. Functionally, awareness of interoceptive signals points out bodily needs, such as the need to drink or to eat, and provides information about one’s emotional state (Calì et al., 2015). Interoceptive accuracy is commonly assessed as the ability to feel one’s own heartbeat in behavioural tasks (Schandry, 1981) while interoceptive sensibility is measured via self-report questionnaires (Garfinkel et al., 2015). Lastly, interoceptive awareness is commonly assessed by comparing interoceptive accuracy (objective) and interoceptive sensibility (subjective) within the same task. Notably, significant age-related declines have been found in both interoceptive accuracy (Khalsa et al., 2009) and sensibility (Khalsa et al., 2009; Murphy et al., 2018).

Indeed, growing evidence identifies interoceptive dysfunction as a significant biomarker for mental disorders in midlife and older adults (Nord and Garfinkel, 2022; Qi et al., 2025). Aging is known to be associated with a decline in interoceptive capacities, with implications for emotional processing (Ulus and Aisenberg-Shafran, 2022) and mental wellbeing (Qi et al., 2025; Zamariola et al., 2019). Lower interoceptive ability has been linked to greater difficulties in verbalizing emotions and mitigating negative affect in daily life (Zamariola et al., 2019), while interoceptive training has been shown to improve emotion regulation and promote mental health. Furthermore, interoception interacts with self-regulatory processes that underlie homeostatic and allostatic control, supporting adaptive emotional and behavioural responses in aging populations (Fazekas et al., 2022; Pfeifer and Cawkwell, 2025; Weiss et al., 2014). These findings highlight that interoception plays a role not only in physiological functioning but also in providing a psychological foundation for active aging, contributing to resilience, emotional stability, and overall quality of life.”

Research suggests that internal bodily signals contribute to shaping how information from different sensory modalities is combined, that is, multisensory integration (Saltafossi et al., 2023, 2025), thereby supporting the construction and maintenance of various forms of body representation. A notable example is the representation of the peripersonal space (Ardizzi and Ferri, 2018), the area immediately surrounding the body, where interactions with objects and other individuals take place. The peripersonal space representation is shaped by multisensory integration (Serino, 2019), as the brain combines visual, tactile, and proprioceptive inputs to establish a safety margin for action and interaction. During aging, disruptions in multisensory integration can lead to difficulties in spatial orientation and motor coordination (Chepisheva, 2023). In older adults, altered multisensory processes are also associated with an increased risk of falls (Chepisheva, 2023; Mahoney et al., 2019) and diminished multitasking abilities (Kuehn et al., 2018), further impacting overall mobility and daily functioning.

Moreover, there is evidence regarding the contribution of interoception in tactile perception (Al et al., 2020) and sensorimotor abilities, such as mental rotation of body parts (Raimo et al., 2021). Mental rotation of body parts involves the ability to mentally visualize and manipulate the position of one’s own limbs or other body segments, and it is essential for movement planning and interaction with the environment. These processes rely on body image as a foundational internal model of the body’s shape, position, and boundaries. Crucially, body image is shaped not only by external sensory inputs (e.g., visual, and tactile feedback) and motor experiences but also by interoceptive signals, which provide key information about body’s internal states (Ainley et al., 2012). This interplay between interoception and body image (Badoud and Tsakiris, 2017) highlights the importance of internal bodily signals in constructing a coherent and adaptive sense of the self.

Previous research suggests that interoceptive deficits in aging do not only affect internal bodily awareness (Khalsa et al., 2009; Murphy et al., 2018) but also disrupt the integration of multisensory signals (Mahoney et al., 2019). This underscores the importance of interoception as a central mechanism underlying age-related declines in daily functioning. However, while evidence suggests the presence of these connections, systematic investigations into how deficits in interoceptive domains specifically affect multisensory integration, body representation, and, consequently, functional capacity in healthy older adults remain scarce. Given this gap in the literature, further research is needed to better understand the precise role of interoception in age-related declines in functional capacity and overall wellbeing.

The aims of the present study were the following. First, to develop a battery of tests assessing specific aspects of interoceptive states and their awareness, multisensory processes, and exteroceptive body representations. Second, to investigate whether early changes in interoception can reliably predict declines in daily life functioning, potentially interacting with changes in multisensory processes and in exteroceptive body representations. As a result, the most reliable predictors identified through this investigation will guide the selection and refinement of the test battery, ensuring it is both targeted and meaningful.

Tests included in battery were selected based on previous evidence of their relevance to aging-related domains such as body awareness and multisensory integration. However, this is the first systematic attempt to integrate these measures into a single battery designed to predict everyday functioning in healthy older adults. Using a two-step machine learning approach, we examined how interactions between interoception and exteroceptive body representations contribute to daily functioning, aiming to identify predictors that can inform timely, individualized interventions. Tailored to individual needs, such interventions could help slow the early stages of physical decline and support healthy aging.

Given the established link between interoception and emotional processing, we hypothesize that interoceptive measures will predict aspects of daily functioning related to emotional regulation and body perception. Furthermore, we expect that combining interoceptive and exteroceptive measures will enhance the prediction of physical functioning and overall body capacity, providing a more comprehensive understanding of their joint contribution to everyday functioning in older adults.

## 2 Methods and materials

### 2.1 Participants

Based on our previous study, we recruited 60 right-handed, healthy volunteers (40 female; aged 21–87, M = 48.58, SD = 18.17) with an education level ranging from 8 to 24 years (M  = 15.76, SD  =  3.6). Participants were recruited using digital (mailing list, social media) and non-digital (posters) channels. Elder participants were recruited from Uni Tre Associazione Nazionale delle Università della Terza Età (National Association of Universities of the Third Age) – Chieti (Italy). All participants were of Caucasian European descent and resided in Italy. Young adults were also included in the study to ensure a wide interindividual variability in interoceptive and exteroceptive measures across the adult lifespan. This approach allowed us to model continuous rather than categorical age-related patterns and to identify predictors that remain valid throughout adulthood.

Exclusion criteria were: (i) a low score of cognitive function, (ii) history of neurological, psychiatric conditions or any medical condition that could affect cognitive functioning (e.g., Alzheimer’s disease, multiple sclerosis, Parkinson’s disease). Participants self-reported to be in good health, with no neurological or psychiatric disorders, or medical condition that could affect cognitive, mental, or physical function.

Cognitive function was evaluated using Mini-Mental State Examination (MMSE) (Folstein et al., 1975; Magni et al., 1996), the Montereal Cognitive Assessment **(**MoCa) (Carson et al., 2018; Nasreddine et al., 2005), and the Cognitive Estimation Test (CET) (Della Sala et al., 2003; MacPherson et al., 2014; O’Carroll et al., 1994). The cognitive functioning was assessed using a standard cut-off of < 26. A score lower than 26 in MMSE and MoCa possibly indicates impairment in cognitive functions. A full summary of participants’ demographic data, cognitive scores, and values for all experimental variables is provided in Supplementary Table S1.

All participants provided written informed consent. The experimental protocol was approved by the Institutional Ethics Committee at the University “G. d’Annunzio” of Chieti-Pescara (protocol number 23014), and it was performed in compliance with the Declaration of Helsinki’s ethical principles.

### 2.2 Experimental procedure

Eligible participants were scheduled for a laboratory session. During this session, participants completed the Short Form Health Survey (SF-36) (Apolone and Mosconi, 1998; Brazier et al., 1992), a 36-item self-report questionnaire that measures health across eight dimensions, covering functional status, wellbeing, and overall evaluation of health (Brazier et al., 1992). Then, to collect physiological data, participants were asked to rest with their eyes open while sitting on a chair, watching a fixation cross at the centre of a computer screen and letting their mind wander (Raichle et al., 2001). No instructions on breathing were given. During this period, electrocardiogram (ECG) and respiratory signals were simultaneously recorded for 5 min continuously. To this purpose, three ECG electrodes (Ag/AgCl) were placed in a three-lead configuration: two electrodes were positioned on the left side and right side of the participant’s lower abdomen, and another electrode was located underneath the right collarbone; a respiratory effort belt was used to record respiratory signals. The cardiac and respiratory signals were recorded with a BIOPAC MP160 System (BIOPAC System, Inc., Goleta, CA, USA) (low-pass filter: 35 Hz; high-pass filter: 0.05 Hz; notch filter: 50 Hz; sampling rate: 2000 Hz) using the AcqKnowledge software (version 5.0.5, BIOPAC System, Inc., Goleta, CA, USA). Finally, each participant completed a battery of tasks and questionnaires evaluating interoceptive bodily dimensions and different aspects of body representation (Pasciucco et al., 2025b). These measures were selected based on their relevance to bodily awareness and their ability to capture age-related changes in multisensory integration and body perception, as outlined in the study’s primary aims. To avoid response bias related to fatigue the order of task presentation was randomised across participants. A detailed description of all experimental tasks and questionnaires used to assess interoceptive, exteroceptive, and multisensory bodily dimensions is provided in Supplementary Table S1.

### 2.3 Tasks assessing interoceptive bodily dimensions

#### 2.3.1 Interoceptive accuracy

Interoceptive accuracy refers to the accuracy in detecting internal bodily sensations (Garfinkel et al., 2015). In this study we employed two tasks to measure participants’ cardiac interoceptive accuracy: the Heartbeat Detection task (Acc-d), also known as the Tapping or Tracking task (Brener and Ring, 2016; McFarland, 1975), and the Heartbeat Counting task (Schandry, 1981) (Acc-c).

#### 2.3.2 Interoceptive sensibility

Interoceptive sensibility is the subjective account of experiencing internal bodily sensations (Critchley and Garfinkel, 2017). It can be assessed using subjective measures that index both the individual’s confidence in their interoceptive ability and their interoceptive feelings (Garfinkel et al., 2015). We assessed participants’ interoceptive sensibility based on their confidence in interoceptive accuracy during the Heartbeat Detection task (Con-d) and the Heartbeat Counting task (Con-c).

To assess interoceptive sensibility we also used two self-report questionnaires: the Body Perception Questionnaire-22 (BPQ) (Poli et al., 2021; Porges, 1993) and the Multidimensional Assessment of Interoceptive Awareness (MAIA) (Calì et al., 2015; Mehling et al., 2012).

The BPQ consists of 22 items and a three-factor structure including a body awareness factor, a supradiaphragmatic factor, and a subdiaphragmatic/body awareness factor. The body awareness (BOA) factor consists of items related to the upper parts of the body, the supradiaphragmatic (SUP) involved in regulating of the functions of organs situated above the diaphragm and additionally, the subdiaphragmatic/body awareness factor (BOA/SUB), which includes items related to subdiaphragmatic issues. The MAIA is composed of 32 items and eight subscales: M1 Noticing, the awareness of one’s body sensations; M2 Not-distracting, the tendency not to ignore or distract oneself from sensations of pain or discomfort; M3 Not-worrying, the tendency not to experience emotional distress or worry with sensations of pain or discomfort; M4 Attention regulation, the ability to sustain and control attention to body sensation; M5 Emotional awareness, the awareness of the connection between body sensations and emotional states; M6 Self-regulation, the ability to regulate psychological distress by attention to body sensations; M7 Body listening, the tendency to actively listen to the body for insight; and M8 Trusting: the experience of one’s body as safe and trustworthy.

#### 2.3.3 Interoceptive awareness

We assessed participant’s interoceptive awareness comparing the accuracy and the confidence in both the Heartbeat Detection task (Aw-d) and the Heartbeat Counting task (Aw-c).

### 2.4 Tasks assessing exteroceptive bodily dimensions

#### 2.4.1 Body image

Body image refers to the subjective experience of the physical structure of our body in terms of its size, shape, and physical composition (Longo, 2016). We assessed body image through the Photographic Figure Rating Scale (PFRS) task, adapted from Naor-Ziv et al. (2020). This task was used to assess participants’ body image perception and body shape dissatisfaction.

#### 2.4.2 Spatial tactile acuity

Spatial tactile acuity refers to the ability to precisely perceive the location and the quality of touch (Harvie et al., 2018). This dimension was evaluated through the Two-point discrimination (2PD) task, which assesses participants’ ability to distinguish two closely spaced points on a small area of the skin and the accuracy of their discrimination abilities (Weber, 1996).

#### 2.4.3 Body structural representation

Body structural representation refers to the knowledge about the topological organization of one’s own body, outlining how different body parts interrelate within a spatial configuration, focusing on the spatial positioning of each body part in relation to others (Longo, 2016). This dimension was evaluated through the Finger Localization task (FLT) (Benton et al., 1983), which requires participants to identify and differentiate the fingers stimulated in three different conditions.

#### 2.4.4 Multisensory integration

Multisensory integration refers to the process by which inputs from two or more sensory modalities are combined by the nervous system to form a stable and coherent percept of the world (Yau et al., 2015). This dimension was evaluated through the Multisensory Integration (MSI) task aiming at investigating the integration of visual, auditory, and tactile stimuli in participants’ perception (Mahoney and Verghese, 2019).

#### 2.4.5 Multisensory temporal resolution

Multisensory temporal resolution refers to the principle that optimal multisensory integration occurs when stimuli from different sensory modalities are presented closely in time. This principle highlights the critical role of temporal proximity in the integration of sensory information from different modalities (Sarko et al., 2012). This dimension was evaluated through the Simultaneity Judgment (SJ) task, which aims to measure temporal sensitivity in the integration of multisensory stimuli.

#### 2.4.6 Peripersonal space

Peripersonal space refers to the space surrounding one’s own body, where the integration of stimuli on the body and from the external environment is facilitated (Rabellino et al., 2020). We evaluated this dimension through the Peripersonal Space (PPS) task (Canzoneri et al., 2012; Di Cosmo et al., 2018, 2021), which measures individual peripersonal space boundaries by assessing the optimal temporal interval for the integration of tactile and auditory stimuli (Ferri et al., 2015; Spadone et al., 2021).

#### 2.4.7 Temporal tactile acuity

Temporal tactile acuity is the ability to detect and distinguish temporal characteristics of sensations related to touch (Laasonen et al., 2001). We assessed this dimension through the Temporal Order Judgment (TOJ) task which evaluate the capacity to detect and distinguish temporal characteristics of sensations related to touch.

#### 2.4.8 Sensorimotor functions

##### 2.4.8.1 Touch remapping

To perceive the location of touch in space, the brain combines information about touched skin location with information about the location of that body part in space. When the two hands are in a “crossed” position, this integration is impaired, affecting the ability to judge the order of touches on both hands (Azañón et al., 2016), creating a conflict between how tactile senses represent external space. Consequently, the same cues must be remapped using an external reference frame. This remapping process becomes necessary to accurately judge the temporal order of the tactile stimuli in the crossed-hand condition (Ferri et al., 2016). Participants performed the TOJ task under a crossed-hand condition, crossing their arms over their wrists.

##### 2.4.8.2 Laterality judgment task

The Laterality Judgement Task (LJT) is designed to assess participants’ ability to mentally rotate hand images and accurately judge the lateral orientation of the hand images (Mibu et al., 2020). We employed this task to assess the efficiency of the neural mechanism underlying the mental rotation process.

## 3 Data analysis

### 3.1 Physiological measurements

ECG data were processed using MATLAB (R2021b, MathWorks Inc., Natick, MA, USA). R-peaks were detected using the Pan-Tompkins algorithm (Pan and Tompkins, 1985). To measure Heart Rate Variability (HRV), the physiological phenomenon of variation in the time interval between heartbeats, the time series of the distance between consecutive R-peaks (tachogram) was computed. We used Kubios software (Kubios HRV Scientific 4.1.0) (Tarvainen et al., 2014) to pre-process the tachogram and extract mean heart rate (HR) and HRV frequency-domain features of interest. The ECG tachogram was interpolated at 4 Hz. Frequency-domain analysis used the Fast Fourier Transform-based Welch’s periodogram applied to the detrended RR series. We calculated mean HR, high frequency (HF:0.15–0.4 Hz) log power (log ms2/Hz, the log-transformed power of HF band) and low frequency/high frequency ratio (LF/HF).

Respiratory data were processed using MATLAB. Respiratory phases (inhale and exhale) were detected following a validated procedure described by Grund et al. (2022), and each respiratory cycle was defined as the interval from one inhalation onset to the subsequent one. Finally, breath frequency was calculated as the number of breaths per minute.

Mean HR, HF power, LF/HF, and breath frequency were included as physiological variables in the model.

### 3.2 Measurements of interoceptive bodily dimensions

#### 3.2.1 Interoceptive accuracy

To calculate accuracy in the Heartbeat Detection task (Acc-d), the recorded R-peaks and the corresponding tapping responses were compared (Fittipaldi et al., 2020; García-Cordero et al., 2017; Yoris et al., 2017). The tapping responses were categorized based on participant’s HR (Fittipaldi et al., 2020; García-Cordero et al., 2017; Yoris et al., 2017). Three categories were used: HR < 69.75, 69.75 < HR < 94.25, and HR > 94.25. For each heartbeat, the difference between the time of the tapping and the nearest R-peak was calculated. If the HR lay into one of the predefined categories, the tapping response within a specific time window was considered correct. The number of correct tapping responses was tallied for each HR category. The total correct responses were denoted as corr_69 (HR < 69.75), corr_betw (69.75 < HR < 94.25), and corr_94 (HR > 94.25). The task accuracy was then calculated with the following formula, as presented by Zaccaro et al. (2022):

accuracy = 1 - (|recorded heartbeats - (corr_69 + corr_betw + corr_94)| / recorded heartbeats).

As for the Heartbeat Counting Task, the accuracy (Acc-c) was calculated by comparing the recorded heartbeats and the counted heartbeats (Koch et al., 2014). Participants’ interoceptive accuracy scores were computed according to the following formula (Schandry, 1981):

accuracy = 1/4 *Σ* [1 − (|recorded heartbeats − counted heartbeats| / recorded heartbeats)].

#### 3.2.2 Interoceptive sensibility

We evaluated interoceptive sensibility through confidence rating in interoceptive accuracy during the Heartbeat Detection task (Con-d). The confidence was assessed using a 9-point scale ranging from 1, indicating low confidence (total guess/no heartbeat awareness), to 9, indicating high confidence (complete perception of heartbeat). The final confidence score was calculated by averaging the ratings in the two trials.

A similar procedure was used to evaluate the level of confidence during the Heartbeat Counting task (Con-c); the confidence score was calculated by averaging the ratings in the four trials.

Interoceptive sensibility was further assessed through two self-report measures: the Body Perception Questionnaire (BPQ) (Poli et al., 2021; Porges, 1993) and the Multidimensional Assessment of Interoceptive Awareness (MAIA) (Calì et al., 2015; Mehling et al., 2012).

#### 3.2.3 Interoceptive awareness

Interoceptive awareness was calculated comparing the objective accuracy with the subjective confidence for both Heartbeat Detection task (Aw-d) and Heartbeat Counting task (Aw-c), calculating the absolute difference between rescaled (min-max normalization) measures of interoceptive accuracy and confidence (Mattioni et al., 2024).

### 3.3 Measurements of exteroceptive bodily dimensions

#### 3.3.1 Body image

The height and weight of each participant were used to calculate the real body mass index, BMI (R), with the standard formula: BMI = weight (kg) / [height (m)]^2^. Based on participants’ rankings on the PFRS, we calculated the mean BMI for participant’s perceived actual physique, indicated as BMI (A). These means were derived from the participants’ ratings of the silhouette images on the PFRS. Lastly, we calculated the body image (ΔAR) as the difference between the participants’ mean BMI (A) and the BMI (R).

#### 3.3.2 Spatial tactile acuity

We calculated the accuracy in the Two Point discrimination (2PD) task as percentages of trials in which participants provided correct responses for each condition and included the score of correct responses on chest (C-che) and arm (C-arm).

#### 3.3.3 Body structural representation

Considering the third condition of the FLT, we calculated the accuracy and included the score of correct responses (HIT) as a variable.

#### 3.3.4 Multisensory integration

To analyse the data collected with the MSI, first the RTs were organized by sorting them according to the experimental condition: Auditory (A), Tactile (T), Visual (V), Audio-Tactile (AT), Audio-Visual (AV), Visuo-Tactile (VT). Each trial was represented in a separate row, with the corresponding RT recorded in the cells. In the next step, the cumulative distribution frequency (CDF) was calculated for each experimental condition, which involved determining the proportion of RTs falling within each time bin. The CDF values were then summed across the time bins to create a cumulative probability distribution for each condition. Finally, the actual and predicted CDFs were compared to evaluate the presence of multisensory integration. This was done by subtracting the predicted CDF, obtained by summing the individual unimodal CDFs, from the actual CDF. Positive values indicated integration of the unimodal stimuli and a violation of the Race Model Inequality. The analysis also involved calculating the area-under-the-curve (AUC) for the violated percentile range to quantify the magnitude of multisensory integration (Mahoney and Verghese, 2019).

#### 3.3.5 Multisensory temporal resolution

We calculated the JND ‘just noticeable difference’ from Simultaneity Judgment (JND-sj): this parameter reflects the subject’s sensitivity to changes in temporal intervals between the stimuli. The JND value denotes the minimal temporal interval at which the change between the perceived temporal relation stimuli can be observed (Binder, 2015). The responses were analysed using a Gaussian distribution to obtain the JND-sj.

#### 3.3.6 Peripersonal space

To estimate the individual boundary of the PPS, we analysed the RTs with the Spearman–Karber method to estimate the point of subjective equality (PSE-pps) of the psychometric function (Miller and Ulrich, 2001). This method involves fitting a psychometric function to the RTs, which relates the probability of a correct response to tactile distance. The PSE is then obtained as the midpoint of the psychometric function and is taken as a proxy of the peripersonal space boundary. This method has been widely used to estimate sensory thresholds and perceptual boundaries in various experimental contexts (Ferroni et al., 2022; Masson et al., 2021).

#### 3.3.7 Temporal tactile acuity

From uncrossed hand condition of TOJ, we measured the JND (JND-toju), which represents the smallest interval at which participants can reliably determine which of the two presented sensory inputs was presented first (Kostaki et al., 2018). Participant responses were calculated by considering the temporal distance between stimuli (SOA). To analyse the data, a fitting function was applied using a normal distribution model (Ferri et al., 2016).

#### 3.3.8 Sensorimotor functions

##### 3.3.8.1 Touch remapping

From the TOJ condition with crossed hands, we measured the JND (JND-tojc), which represents the smallest interval at which participants can reliably determine which of the two presented sensory inputs came first. Participant responses were calculated from the SOAs. To analyse the experimental data, a fitting function was applied using a normal distribution model (Ferri et al., 2016).

To quantify the overall difference between the crossed and uncrossed hands conditions, the cumulative sum of differences between the fitted models was computed. This yielded a global indicator of difference, denoted as “sum of confusion” (SC), providing an overarching measure of the divergence between the two conditions (Ferri et al., 2016; Wada et al., 2014).

##### 3.3.8.2 Laterality judgment task

In LJT both accuracy and response speed are essential performance measures. We calculated slopes, which reflect the efficiency of the neural mechanism underlying the mental rotation process: a smaller slope indicates higher neural efficiency in mental rotation (Christova et al., 2008). We quantified slopes for both hands, indicated as Mental Rotation Efficiency for the left hand (MRE-lh) and the right hand (MRE-rh).

## 4 Statistical analysis: machine learning approach

We adopted a two-step machine learning approach to investigate the contributions of interoceptive dimensions and body representations to daily functioning, and to examine their interplay in predicting individual outcomes. The goal was to identify the most predictive interoceptive measures and assess whether including body representation variables enhances prediction by integrating internal and external bodily signals. Partial Least Squares Regression (PLSR) was employed as a dimensionality-reduction technique effective in addressing multicollinearity and minimizing overfitting. PLSR builds regression models by projecting predictors into a lower-dimensional space of uncorrelated components, linear combinations of the original variables, thus capturing the most informative variance while reducing model complexity.

We implemented leave-one-out nested cross-validation (nCV) to optimize the number of components (K) and ensure generalizability. The SF-36 subscale scores were used as dependent variables: Physical Functioning, Role Limitations (Physical and Emotional), Energy/Fatigue, Emotional wellbeing, Social Functioning, Bodily Pain, and General Health Perceptions.

In the first step, we trained eight separate PLSR models (one per SF-36 domain) using the following interoceptive predictors: Acc-d, Acc-c, Con-d, Con-c, BOA, SUP, BOA/SUB, 1 M–8 M, Aw-d, Aw-c, Breath Frequency, Mean HR, HF, and LF/HF. Each model was independently tested to identify the most relevant predictors for each outcome.

In the second step, PLSR models were constructed only for those SF-36 dimensions significantly predicted in the first step. The significant interoceptive predictors were combined with body representation measures, including: body image (ΔAR), tactile acuity (C-che, C-arm), body structural representation (HIT), multisensory integration (AUC-av, AUC-at, AUC-vt), multisensory temporal resolution (JND-sj), peripersonal space (PSE-pps), temporal tactile acuity (JND-toju), and sensorimotor function (JND-tojc, SC, MRE-lh, MRE-rh).

## 5 Results

### 5.1 Step 1: predictive impact of interoceptive dimensions to daily functioning

To explore whether and to what extent different daily functioning dimensions are influenced by interoceptive dimensions we ran eight models, one for each SF-36 subscale.

**Figure 1:**
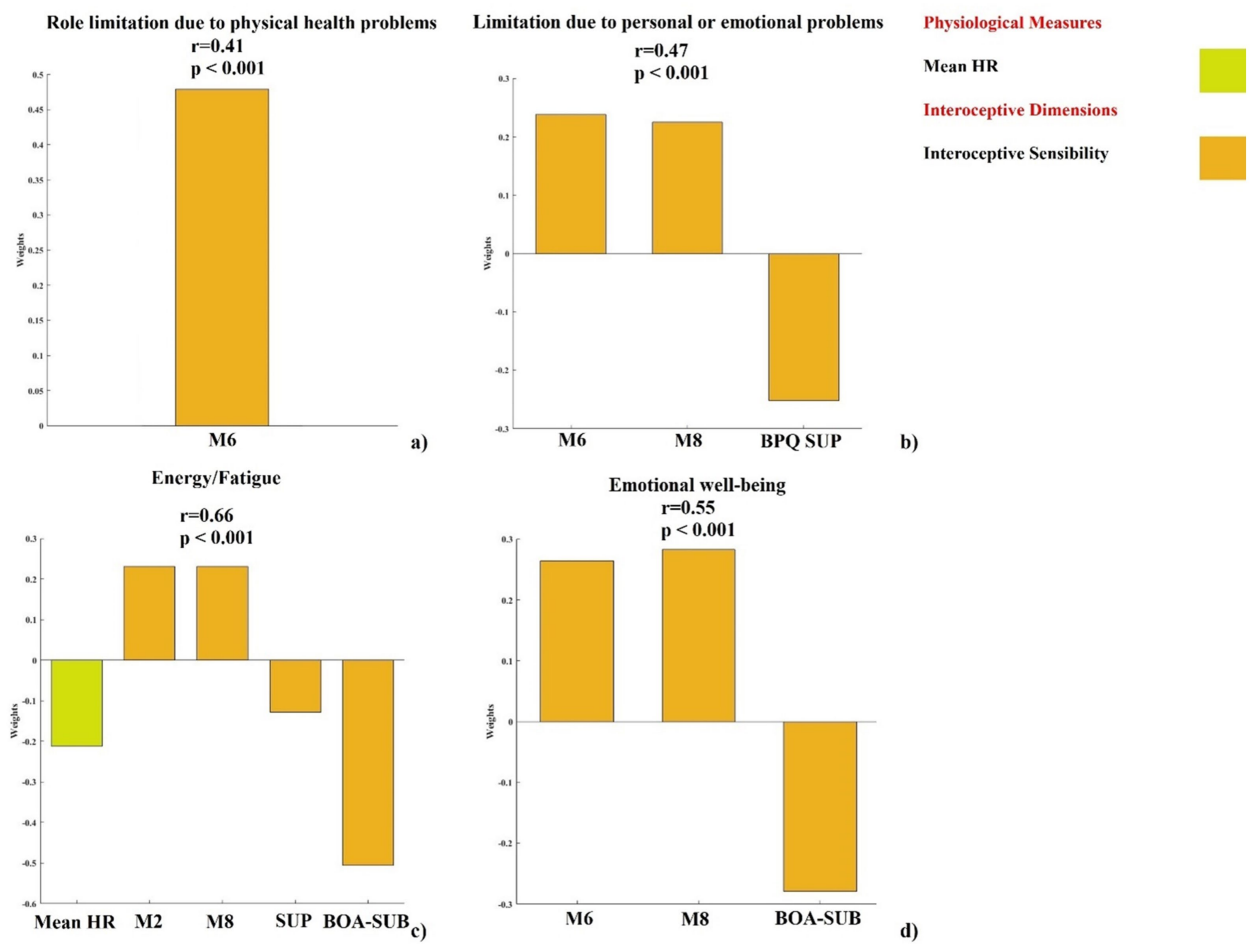
Contribution of interoceptive dimensions to SF-36 subscales. PLSR out-of-sample prediction of SF-36 subscales shows significant correlations between true and predicted values (*r* and *p-*values reported). **(a)** Role limitations due to: regression weights for interoceptive variables (M6). **(b)** Role limitations due to personal or emotional problems I: weights for variables (M6, M8, BPQ-SUP). **(c)** Energy/fatigue: weights for variables (mean HR, M2, M8, BPQ-SUP, BPQ-BOA/SUB). **(d)** Emotional wellbeing: weights for variables (M6, M8, BPQ-BOA/SUB). Only weights exceeding the 95% confidence interval of the null hypothesis are reported. BOA/SUB = subdiaphragmatic/body awareness factor of BPQ; BPQ SUP = supradiaphragmatic factor of BPQ; M2 = Not-Distracting MAIA scale; M6 = Self-Regulation MAIA scale; M8 = Trusting MAIA scale; Mean HR = mean heart rate.

We found that the model testing the predictive impact of interoceptive dimensions on Role Limitations due to physical health problems scale was significant after cross-validation (see Figure 1a). A significant positive correlation was found between the actual and the predicted scores in Role Limitations due to physical health problems subscale (*r* = 0.41, *p* < 0.001). The only dimension that significantly contributed was interoceptive sensibility, specifically the MAIA 6 M subscale.

The model testing the predictive impact of interoceptive dimensions on Role Limitations due to personal or emotional problems scale was significant after cross-validation (see Figure 1b). A significant positive correlation was found between the actual and the predicted Role Limitations due to personal or emotional problems subscale (*r* = 0.47, *p* < 0.001). The only dimensions that significantly contributes was interoceptive sensibility, specifically the MAIA 6 M and 8 M subscales, and the BPQ-SUP subscale.

A significant association between the predictive impact of interoceptive dimensions on the Energy/fatigue scale emerged from the model after cross-validation (see Figure 1c). A significant positive correlation was found between the actual and the predicted Energy/fatigue subscale (*r* = 0.66, *p* < 0.001). In this instance, a physiological contribution, specifically the mean HR, worked in synergy with contributions from various aspects of interoceptive sensibility, specifically MAIA 2 M and 8 M subscales and BPQ-SUP and BPQ-BOA/SUB subscales.

The PLSR analysis revealed that interoceptive dimensions significantly contributed to the prediction on Emotional wellbeing scale (see Figure 1d). We found a significant positive correlation between the actual and the predicted Emotional wellbeing subscale (*r* = 0.55, *p* < 0.001). Interoceptive sensibility, specifically MAIA 6 M and 8 M subscales, and BPQ-BOA/SUB emerged as the key aspects of interoceptive sensibility that significantly contributed.

The models testing the predictive impact of interoceptive dimensions on SF-36 subscales Physical Functioning, Social Functioning, Bodily pain and General Health perceptions were non-significant after cross-validation.

### 5.2 Step 2: predictive impact of the interaction between interoceptive dimensions and body representation on daily functioning

We evaluated how the inclusion of body representation variables, interacting with interoceptive measures, contributes to the prediction of daily functioning outcomes for the SF-36 subscales that were significantly predicted by interoceptive dimensions in Step 1. Specifically, we aimed at identifying which exteroceptive body representation measures, in combination with interoceptive measures, emerge as significant predictors. Hence, we ran four separate PLSR models, one for each of the following SF-36 subscales: Role Limitations due to physical health problems, Role Limitations due to personal or emotional problems, Energy/fatigue and Emotional wellbeing.

For each model, the significant interoceptive predictors identified in the first step were retained: mean HR, 2 M, 6 M, 8 M, BPQ-SUP and BPQ-BOA/SUB. In addition, body representation measures were included: ΔAR, C-che, C-arm, HIT, AUC-av, AUC-at, AUC-vt, JND-sj, PSE-pps, JND-toju, JND-tojc, SC, MRE-lh, MRE-rh.

**Figure 2:**
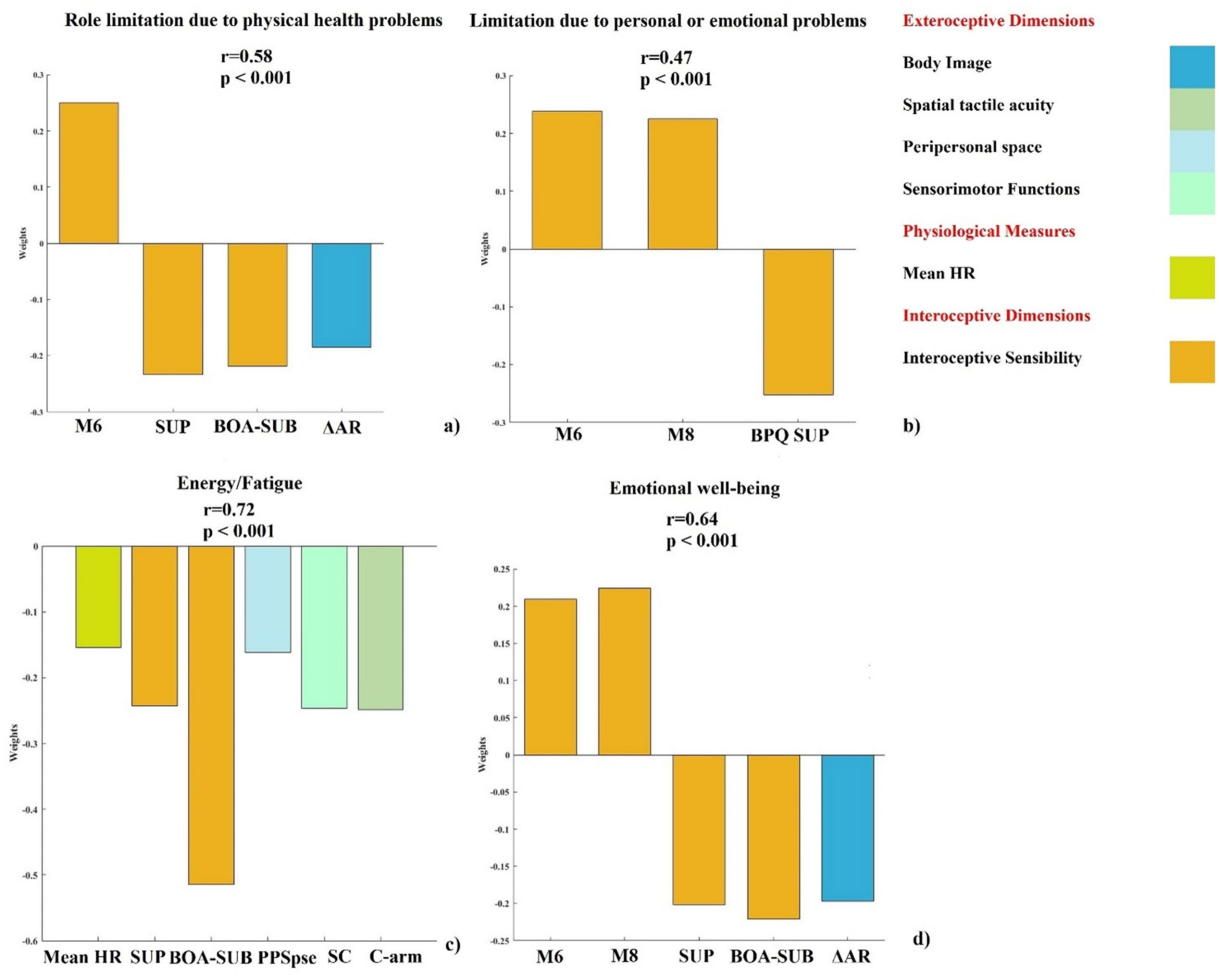
Contribution of significant interoceptive dimensions and body representation variable to selected SF-36 subscales. PLSR out-of-sample prediction of SF-36 subscale shows significant correlations between true and predicted values (*r* and *p* values reported). **(a)** Role limitations due to physical health problems: regression: weights for variables (M6, BPQ-SUP, BPQ-BOA/SUB, ΔAR). **(b)** Role limitations due to personal or emotional problems: weights for variables (M6, M8, BPQ-SUP). **(c)** Energy/fatigue: weights for variables (Mean HR, BPQ-SUP, BPQ-BOA/SUB, PSE-pps, SC, C-arm). **(d)** Emotional wellbeing: weights for variables (M6, M8, BPQ-SUP, BPQ-BOA/SUB, ΔAR). Only weights exceeding the 95% confidence interval of the null hypothesis are reported. BOA/SUB = subdiaphragmatic/body awareness factor of BPQ; BPQ SUP = supradiaphragmatic factor of BPQ; M6 = Self-Regulation MAIA subscale; M8 = Trusting MAIA subscale; Mean HR = mean heart rate; PSE-pps = point of subjective equality in PPS task; C-arm = correct response arm condition in 2PD task; ΔAR = discrepancies between BMI (A) and the participant’s BMI (R); SC = sum of confusion in TOJ task.

The model testing the predictive impact of interoceptive dimensions and body representation variables on Role Limitations due to physical health problems subscale was significant after cross-validation (see Figure 2a). We found a significant positive correlation between the actual and the predicted Role Limitations due to physical health problems subscale (*r* = 0.58, *p* < 0.001). The dimensions that significantly contributed were: interoceptive sensibility (6 M, BPQ-SUP, BPQ-BOA/SUB) and body image (ΔAR).

The PLSR model confirmed that interoceptive dimensions remained significant predictors on Role limitations due to personal or emotional problems subscale after cross-validation (see Figure 2b). A significant positive correlation between the actual and the predicted Role Limitations due to personal or emotional problems subscale was found (*r* = 0.47, p < 0.001). The variables that significantly contributed were: 6 M, 8 M and BPQ-SUP. Notably, in this model, the same interoceptive sensibility measures were confirmed as significant predictors of Role Limitations due to personal or emotional problems. No exteroceptive body representation variables emerged as significant predictors, suggesting that interoceptive dimensions alone were sufficient to explain this aspect of daily functioning.

The model testing the predictive impact of interoceptive dimensions and body representation variables on Energy/fatigue subscale was significant after cross-validation (see Figure 2c). A significant positive correlation between the actual and the predicted Energy/fatigue subscale and the predicted was observed (*r* = 0.72, *p* < 0.001). The dimensions that significantly contributed were physiological measures (mean HR), interoceptive sensibility (BPQ-SUP, BPQ-BOA/SUB), peripersonal space (PSE-pps), sensorimotor function (SC), and spatial tactile acuity (C-arm).

The model testing the predictive impact of interoceptive dimensions and body representation variables on Emotional wellbeing subscale was significant after cross-validation (see Figure 2d). A significant positive correlation between the actual and the predicted Emotional wellbeing subscale was found (*r* = 0.64, *p* < 0.001). The dimensions that significantly contributed were: interoceptive sensibility (6 M, 8 M, BPQ-BOA/SUB) and body image (ΔAR).

The dimensions that significant predict Role Limitations due to physical health problems, Role Limitations due to personal or emotional problems, Energy/fatigue, and Emotional wellbeing are summarized in Table 1.

**Table 1:** Dimension that contributes significantly to role limitations due to physical health problems, role limitations due to personal or emotional problems, energy/fatigue, and emotional wellbeing.

	Role limitations due to physical health problems	Role limitations due to personal or emotional problems	Energy/fatigue	Emotional wellbeing
Exteroceptive dimensions	Body image (AAR)		Spatial tactile acuity (C-arm) peripersonal space (PSE-pps) sensorimotor functions (SC)	Body image (AAR)
Physiological measures			Mean HR	
Interoceptive dimensions	Interoceptive sensibility (M6, BPQ-SUP, BPQ-BOA/SUB)	Interoceptive sensibility (M6, M8, BPQ-SUP)	Interoceptive sensibility (BPQ-SUP, BPQ-BOA/SUB)	Interoceptive sensibility (M6, M8, BPQ-SUP, BPQ-BOA/SUB)

## 6 Discussion

The aim of the present study was twofold. First, to develop a comprehensive battery of tests designed to assess specific aspects of interoceptive states and their awareness, multisensory integration, and exteroceptive body representations. Second, to examine whether early alterations in interoceptive functioning can reliably predict declines in daily life activities, potentially in interaction with changes in multisensory processing and exteroceptive body representation. The most robust predictors identified through this investigation informed the selection and refinement of the test battery.

Tests and questionnaires assessing interoception, multisensory processes and exteroceptive body representations were selected based on prior evidence of their relevance in predicting changes in daily life functioning in healthy older population. We then used these tests to collect measures among 60 healthy subjects, including both young, middle-aged and elderly subjects. Although the sample covered a broad age range (21–87 years), the study primary aim was to identify mechanisms that are particularly relevant to healthy aging. The inclusion of younger adults served to increase variability and capture the full continuum of interoceptive and multisensory functioning across adulthood. In this view, age was not considered as an independent variable, but as the broader context within which bodily awareness and multisensory integration gradually change and acquire their adaptive or compensatory meaning. We used the collected data to assess whether early changes in interoception, potentially interacting with multisensory processes and exteroceptive body representations, could reliably predict declines in daily functioning. To achieve this, we employed the PLSR, a robust machine learning approach to examine the interactions between multiple predictors and outcomes, to evaluate the contribution of each of the collected measures. The procedure included two steps: first, we focused on the predictive role of interoceptive bodily dimensions, including interoceptive accuracy, sensibility, and awareness. Second, we combined relevant interoception dimensions, as emerged from the first step, with multisensory processes and exteroceptive body representations measures to understand whether their interaction influences active aging.

The approach employed in this study is particularly relevant. It underscores the predictive power of specific interoceptive and exteroceptive measures but also provides a foundation for developing timely, individualized interventions tailored to the needs and characteristics of older adults, with the aim of slowing down the initial stages of physical decline and promoting healthy aging.

### 6.1 Predictive role of interoceptive dimensions to daily functioning

In the first step of the machine learning analysis, we explored the predictive role of interoceptive bodily dimensions in daily functioning. The results indicate that interoceptive sensibility, measured by different MAIA and BPQ subscales, significantly predicts the following aspects of daily functioning, measured by the SF-36 questionnaire: Role Limitations due to physical health problems, Role Limitations due to personal or emotional problems, Energy/fatigue, and Emotional wellbeing.

These results support the hypothesis that interoceptive sensibility is a crucial predictor of functioning in older adults and underscore the importance of reevaluating the role of interoception in aging: the subjective evaluation of bodily signals seems more relevant to daily function than objective detection accuracy. These results are consistent with three main interpretative frameworks that highlighted the importance of interoceptive sensibility in maintaining functional capacity and emotional regulation in aging populations (Carstensen et al., 2011; Kunzmann et al., 2000; Pfeifer and Cawkwell, 2025). First, from a compensatory perspective, interoceptive sensibility and in particular self-regulation—the perception of the body as safe, and the awareness of bodily states—may counterbalance age-related declines in interoception by enhancing subjective monitoring of bodily and emotional functions. These aspects of interoceptive sensibility are involved in emotional wellbeing and self-regulation (Carstensen et al., 2011; Kunzmann et al., 2000), while the perception of the body as a safe space may promote emotional stability (Price and Hooven, 2018) and reduce the impact of emotional distress on daily functioning. Second, drawing on embodied cognition models of aging (Pfeifer and Cawkwell, 2025), physiological and sensory decline can impair the integration of exteroceptive and interoceptive signals, affecting body representation and sense of self (Costello and Bloesch, 2017; Kuehn et al., 2018). Age-related declines in interoceptive accuracy may disrupt the coherence of these multisensory processes (Monti et al., 2021). Yet, the ability to trust one’s own body and regulate emotions through bodily awareness may serve as a buffer, helping to preserve emotional resilience in the face of these structural changes. Third, the findings are consistent with the Socioemotional Selectivity Theory, which posits that as people get older and perceive their remaining time as limited, they increasingly prioritize emotionally meaningful and low-arousal experiences (Carstensen et al., 1999). According to this theory, older adults may rely on bodily awareness as a strategy to regulate distress and enhance emotional focus, further reinforcing their preference for emotionally salient states that support wellbeing.

In more detail, the self-regulation (M6), which refers to ability to regulate distress by attending to bodily sensations, emerged as a key predictor of physical role limitations. This suggests that the ability to regulate distress through attention to bodily sensations may influence individuals’ perceptions of physical limitations and help older adults more effectively manage physical discomfort. In turn, this may support more adaptive responses to physical activities (Wallman-Jones et al., 2021) and potentially reduce the limitations in daily functioning. Moreover, this regulatory ability may also enhance performance in physical tasks (Wallman-Jones et al., 2021), even in the face of natural age-related physical changes (Pfeifer and Cawkwell, 2025). In addition to self-regulation, both the ability to perceive the body as safe and trustworthy (M8) and the awareness of internal states of the body (BPQ-SUP) significantly predicted emotional role limitations and emotional wellbeing. These findings underscore the central role of bodily awareness in modulating emotional states in everyday life and in promoting affective stability. Moreover, these results reinforce the established link between interoception and emotional regulation (Barrett, 2017; Feldman et al., 2024). The integration of interoceptive signals with emotional processes is crucial for maintaining emotional wellbeing. Research indicates that interoceptive sensibility improves emotional regulation by enabling individuals to accurately interpret and respond to bodily signals associated with emotional states (Füstös et al., 2013; Price and Hooven, 2018). In older adults, this integration may be particularly crucial, as age-related declines in interoceptive accuracy may weaken the connection between bodily sensations and emotional experiences (MacCormack et al., 2023; Mendes, 2010), potentially compromising emotional resilience.

The subjective experience of energy levels and fatigue (Energy/fatigue scale) was predicted by heart rate frequency and several aspects of interoceptive sensibility, including the ability to not distract oneself from sensation of pain or discomfort (M2), the perception of the body as safe and trustworthy (M8) and the awareness of bodily states (BPQ-SUP, BPQ-BOA/SUB). Cardiac functioning holds a measurable relationship with fatigue (Nelesen et al., 2008) and HR is an important indicator of physical effort and energy expenditure, as it positively correlates with perceived fatigue (Nelesen et al., 2008). Therefore, HR may serve as a physiological marker of fatigue, particularly when individuals experience prolonged or intense physical demands. Previous research has shown that HR plays a role in maintaining energy levels and reducing fatigue (Thayer et al., 2012). Specifically, resting cardiac autonomic balance, characterized by parasympathetic dominance over sympathetic influences, favours energy conservation and recovery. The role of cognitive fatigue in influencing physical performance has been further emphasized, describing fatigue as a brain-derived emotion that regulates exercise behaviour to ensure the protection of whole-body homeostasis (Marcora et al., 2009). Our findings align with this perspective, suggesting that interoceptive sensibility may play a key role in modulating the perception of fatigue by integrating bodily signals with cognitive and emotional processes (McMorris, 2020). More in detail, the ability to attend to and trust bodily sensations may help individuals to better manage cognitive fatigue, thereby maintaining energy levels and physical performance even during physiological challenges. Additionally, the ability to not distract from sensation of pain or discomfort may reflect a capacity to tolerate discomfort, which could be crucial for sustaining effort during physically demanding tasks. This suggests that both physiological markers, such as HR, and interoceptive sensibility contribute to the perception of energy and fatigue levels and their regulation in daily life. These findings underscore the importance of interoceptive sensibility in regulating both cognitive and physical aspects of fatigue.

To summarize, the results of the first step of the analysis indicate that specific interoceptive sensibility sub-domains, as measured by subscales of the MAIA and BPQ questionnaires, play a crucial role in predicting various aspects of daily functioning across adulthood.

Interestingly, interoceptive accuracy and interoceptive awareness did not emerge as significant predictors in this step. This contrasts with some studies that have emphasized the role of objective interoceptive abilities in aging (Khalsa et al., 2009; Murphy et al., 2018). This discrepancy may arise since interoceptive sensibility is a subjective measure that captures a broader range of bodily experiences, which are more directly relevant to daily functioning. As a possible alternative explanation, changes in subjective perceptions of bodily states may become more salient than objective changes during aging. Importantly, our study focuses on several aspects of daily functioning, assessed by different subscales of the SF-36, rather than directly examining age-related changes in interoceptive abilities. This approach allowed us to identify the interoceptive aspects that contribute to specific dimensions of daily functioning, instead of focusing on how interoception declines with age. While age-related changes were not the primary focus of this study, future analysis could assess how subjective and objective interoceptive measures are related to age across the adult lifespan. This distinction may explain why subjective measures of interoception were more predictive than objective ones. Finally, the subjective interoceptive sensibility may compensate for well-documented age-related declines in objective interoceptive accuracy (Khalsa et al., 2009); this could explain why interoceptive accuracy did not emerge as a significant predictor in our study. Therefore, relying on interoceptive sensibility may allow older adults to maintain functional capacity despite physiological changes.

### 6.2 Predictive role of interaction between interoceptive and exteroceptive bodily dimensions to daily functioning

The second step of our analysis aimed to explore whether and how exteroceptive bodily dimensions interact with interoceptive bodily dimensions to predict daily functioning in those SF-36 subscales that were significantly predicted by interoceptive measures in the first step of the analysis. To this purpose, we included in each model several exteroceptive dimensions as potential predictors: body image, spatial tactile acuity, body structural representation, multisensory integration, multisensory temporal resolution, peripersonal space, temporal tactile acuity and sensorimotor functions.

The results revealed that including exteroceptive bodily dimensions enhanced the predictive power of the models for the following dimension of daily functioning: Role Limitations due to physical health problems, Energy/fatigue, and Emotional wellbeing, providing preliminary evidence of an interaction between interoceptive and exteroceptive bodily dimensions in active aging. On the other side, interoceptive dimensions alone were enough to predict outcomes for Role Limitations due to personal or emotional problems, suggesting that aspects of interoceptive sensibility play a central role in this aspect of daily functioning.

Body image (ΔAR) was found to significantly contribute to both limitations due to physical health problems and emotional wellbeing. This suggests that the way individuals perceive their bodies, especially the gap between their actual and the representation of their own body, is closely linked to how they perceive their physical abilities (Alleva and Tylka, 2021) and emotional states (Wilson et al., 2013). These findings support the idea that valuing what the body can do, such as physical abilities, is linked to a more positive body image (Bailey et al., 2015; Frisén and Holmqvist, 2010; Gattario and Frisén, 2019). Additionally, appreciation of physical capacities is associated with interoceptive sensibility, further highlighting its role in shaping how individuals’ perception of their physical capacities (Todd et al., 2019). Moreover, body image plays a critical role in psychological wellbeing, influencing both emotional regulation and self-esteem (Wilson et al., 2013). A positive body image is associated with greater engagement in physical activities and better adherence to healthy behaviours.

Peripersonal space (PSE-pps), spatial tactile acuity (C-arm), and sensorimotor function (SC) were found to predict scores on the Energy/fatigue scale. Although these predictors refer to distinct bodily dimension, they all rely on multisensory integration processes. Indeed, their predictive role supports the idea that reduced efficiency in multisensory processing increases both physical and cognitive effort in everyday tasks. Age-related declines in multisensory processing can impair spatial orientation and motor coordination, making everyday tasks more demanding for older adults. Peripersonal space allows individuals to interact with objects and navigate their surrounding space more efficiently (di Pellegrino and Làdavas, 2015; Serino, 2019). A reduced peripersonal space in older adults may lead to increased cognitive and physical effort when performing daily tasks, such as reaching for objects or avoiding obstacles (Chepisheva, 2023; Ruggiero et al., 2017). This effort can contribute to a faster depletion of energy reserves, leading to higher levels of fatigue (Mahoney et al., 2019). Similarly, spatial tactile acuity, the ability to perceive and discriminate fine spatial details using touch, is essential for efficient movement. This capacity typically declines with age (McIntyre et al., 2021; Stevens and Cruz, 1996), and when both tactile perception and acuity are compromised, individuals may require more cognitive and physical effort to perform basic tasks (Löffler et al., 2024). This increased effort may lead to higher energy expenditure and greater fatigue. Notably, muscle fatigue itself has been shown to increase two-point discrimination thresholds (Han et al., 2015) indicating a further decline in tactile acuity. Sensorimotor function, as measured by SC in TOJ, also reflects the cognitive effort in resolving conflicting sensory information. For example, when the hands are crossed, the brain must remap tactile sensations (Craig et al., 2010; Ferri et al., 2016; Yamamoto and Yamamoto, 2001) to the correct spatial location, a process cognitively demanding. In older adults, who may already experience reduced cognitive reserves, this added processing effort may contribute significantly to feelings of fatigue.

Overall, the findings from the second step of the analysis highlight the importance of integrating body representation measures with interoceptive dimensions to better understand daily functioning in active aging. Our findings are consistent with recent evidence highlighting how the integration of internal and external bodily signals significantly impacts functional and physical activities during aging (Pasciucco et al., 2025a). We found that including exteroceptive factors, such as body image, peripersonal space, spatial tactile acuity and sensorimotor functions in interaction with interoceptive sensibility, significantly contribute to predicting specific aspects of daily functioning: Role Limitations due to physical health problems, Energy/fatigue, also predicted by mean heart rate, and Emotional wellbeing. Taken together, these results provide a more comprehensive model, in which both interoceptive bodily awareness and exteroceptive bodily processing play a critical role in maintaining functional capacity and emotional resilience across adulthood, within an active aging perspective.

The integration observed between interoceptive and exteroceptive bodily dimensions may reflect a broader embodied organization of cognitive and emotional functions. Coordinating internal and external bodily information supports not only perception and action but also the regulation of affective and self-related processes (Kim et al., 2025; Seth, 2013). This view is conceptually consistent with theories of embodiment, which propose that cognitive and affective processes are grounded in the bodily states and sensorimotor systems that support them (Barsalou, 2008; Gallese and Sinigaglia, 2011). Moreover, this perspective is also consistent with models describing a common representational format across sensory domains, where time, space, and magnitude are processed through shared neural codes that enable flexible integration between modalities (Myachykov et al., 2014; Walsh, 2003). Although these theories were originally developed to explain perceptual and cognitive correspondence, the present findings suggest that similar integrative principles may also extend to the interaction between internal and external bodily signals. An example of such cross-modal correspondence may be observed in the association between perceived emotion body maps (Nummenmaa et al., 2014), whereby distinct emotional states are systematically mapped onto specific bodily regions. Importantly, these bodily representations have been shown to undergo developmental changes across childhood (Hietanen et al., 2016), suggesting that embodied emotional representations are not static but evolve across the lifespan. Although empirical evidence in older adulthood is still limited, it is plausible that age-related changes in interoceptive sensitivity and multisensory integration may also affect the structure and functional relevance of these body maps. From this perspective, the present findings suggest that individual differences in the integration of interoceptive and exteroceptive bodily signals may reflect age-related modulations in embodied emotional representations that contribute to everyday functioning in later life. Maintaining this multisensory integration may sustain the sense of the body as a stable reference for action and emotion, a condition described as the body acting as a “safe space.” In line with the Tropic, Embodied, and Situated Theory of cognition (TEST; Myachykov et al., 2014), this experience emerges from the interaction between bodily signals, environmental information, and situational context. Furthermore, according to A Theory of Magnitude (ATOM; Walsh, 2003), interoceptive and exteroceptive dimensions related to safety and wellbeing may rely on shared representational metrics, allowing cross-modal correspondences between internal bodily states and external sensory cues. Age-related modifications in sensory re-weighting mechanisms (Feller et al., 2019; Foisy and Kapoula, 2018; Peterka, 2002) may alter this balance, leading to a greater dependence on external cues, such as visual information, and a reduced reliability of interoceptive and proprioceptive feedback, potentially weakening the perception of the body as a safe space. From this perspective, the present results may capture a functional reorganization of body-related processing with aging, in which the relative weighting of interoceptive and exteroceptive sources contributes to both stability and vulnerability in daily functioning. In this sense, age-related sensory re-weighting may also reflect a shift in spatial reference frames, from egocentric (body-based) to more allocentric or visually anchored representations, consistent with changes in peripersonal space processing.

The present findings also align with contemporary models of frailty, which describe aging-related vulnerability as a multidimensional syndrome involving physical, cognitive, sensory, and psychosocial decline (Cohen et al., 2023; van Oostrom et al., 2017). Within this framework, the combination of interoceptive sensibility and exteroceptive body representations observed in our study may represent embodied correlates of frailty status. Specifically, higher interoceptive self-regulation and body trust could act as protective factors supporting physical and emotional resilience, while alterations in multisensory body processing, such as peripersonal space or tactile acuity, may indicate early stages of functional vulnerability.

From this perspective, our multidimensional battery captures subtle embodied markers that may precede overt manifestations of frailty. These findings suggest that the interaction between interoceptive and exteroceptive bodily dimensions contribute to the maintenance of autonomy and vitality in aging and that its disruption may signal the transition from a “non-frail” to a “pre-frail” condition. Future research could test whether these embodied indicators predict longitudinal changes in frailty status, thereby providing a neurocognitive bridge between body-centered processes and functional health trajectories across adulthood; however, these implications should be interpreted cautiously and validated in older and more diverse cohorts.

### 6.3 Limitations

Despite the promising results, this study presents several limitations. Although the sample include participants with a wide age range, it primarily consisted of healthy individuals with a relatively high levels of education. This also represents a strength for isolating normative lifespan mechanisms in a healthy sample. However, this may limit the generalizability of the results to more diverse populations, particularly in older adults with reduced health conditions or with lower levels of education. Therefore, the strength and the relative contribution of interoceptive and exteroceptive predictors might differ in older adults with frailty-related conditions or lower educational attainment, where variability and constraints on daily functioning are typically greater. The cross-sectional design of the study did not allow to draw causal inferences regarding the relationship between interoceptive dimensions, body representation, and daily functioning. Longitudinal research is needed to explore how these factors interact and evolve across different age stages. Another limitation of this study is the focus on solely cardiac Interoceptive accuracy and awareness. While this is a widely used and well-established domain for studying interoceptive processing, it captures only a single dimension of the broader interoceptive system. Future studies should consider incorporating additional aspects, such as respiratory, gastric, or thermoregulatory signals, to provide a more nuanced and comprehensive understanding of how interoception supports functional and emotional wellbeing in aging populations. Finally, this multidimensional framework could be extended to clinical conditions characterized by altered bodily awareness, multisensory integration, and autonomic regulation, such as Parkinson’s disease, multiple sclerosis anxiety or depression. Investigating interoceptive and exteroceptive predictors in these populations could help clarify how disrupted body–brain coupling contributes to functional decline, emotional dysregulation, and compensatory mechanisms in both neurodegenerative and psychosomatic disorders.

## 7 General conclusions

This study presents a significant advancement in aging research by developing a comprehensive test battery that integrates interoceptive, exteroceptive, and multisensory measures to predict daily functioning in older adults. Unlike the traditional cognitive assessments, this battery highlights the critical role of bodily awareness in aging and offers a practical tool for early identification of functional decline. The findings support its potential use in designing timely, individualized interventions such as mindfulness-based practices, biofeedback training, or multisensory rehabilitation programs to enhance emotional regulation, mobility, and overall quality of life in older populations.

Importantly, this study not only validates the proposed test battery but also empirically refines it, identifying the most predictive and clinically relevant measures, making it more applicable in real-world settings. Future research should focus on validating the battery in more diverse populations, including those with chronic conditions or lower educational backgrounds, and explore digital adaptations to enable remote monitoring and personalized care.

Overall, these findings offer new directions for assessing and supporting active aging through evidence-based, body-focused approaches.

## Data Availability

The raw data supporting the conclusions of this article will be made available by the authors, without undue reservation.

## Glossary

A: Auditory
ΔAR (A-R): discrepancies between BMI (A) and the participants BMI (R)
Acc-c: Accuracy in heartbeat counting task
Acc-d: Accuracy in heartbeat detection task
AT: Audio-Tactile
AUC: Area under the curve
AV: Audio-Visual
Aw-c: Awareness in heartbeat counting task
Aw-d: Awareness in heartbeat detection task
BMI (R): Body Mass Index (Real: BMI = weight (kg) / [height (m)]^2^)
BMI (A): Actual (refers to Actual BMI)
BPQ: Body Perception Questionnaire
BPQ-BOA: Body Perception Questionnaire - Body Awareness subscale
BPQ-BOA/SUB: Body Perception Questionnaire - Body Awareness/Subdiaphragmatic reactivity subscale
BPQ-SUP: Body Perception Questionnaire - Supradiaphragmatic Reactivity subscale
C-arm: Correct response for arm
C-che: Correct response for chest
CDF: Cumulative Distribution Frequency
CET: Cognitive Estimation Test
Con-c: Confidence in heartbeat counting task
Con-d: Confidence in heartbeat detection task
ECG: Electrocardiogram
FLT: Finger Localization Task
HF: High Frequency
HIT: Correct response to FLT
HR: Heart Rate
HRV: Heart Rate Variability
JND: Just Noticeable Difference
LF: Low Frequency
LF/HF: Ratio between low frequency and high frequency
LJT: Laterality Judgment Task
MAIA: Multidimensional Assessment of Interoceptive Awareness
M1: Noticing MAIA scale
M2: Not-Distracting MAIA scale
M3: Not-Worrying MAIA scale
M4: Attention Regulation MAIA scale
M5: Emotional Awareness MAIA scale
M6: Self-Regulation MAIA scale
M7: Body Listening MAIA scale
M8: Trusting MAIA scale
MMSE: Mini-Mental State Examination
MoCa: Montereal Cognitive Assessment
MRE: Mental Rotation Efficiency
MSI: Multisensory Integration
PFRS: Photographic Figure Rating Scale
PLRS: Partial Least Squares Regression
PPS: Peripersonal Space
PSE: Point of Subjective Equality
RT: Reaction Time
SC: Sum of Confusion
SF-36: Short Form Health Survey
SJ: Simultaneity Judgment
SOA: Stimulus Onset Asynchrony
T: Tactile
TOJ: Temporal Order Judgment
V: Visual
VT: Visuo-Tactile

## References

[ref1] Ageing and Health. (2025). Available online at: https://www.who.int/news-room/fact-sheets/detail/ageing-and-health (Accessed April 15, 2025)

[ref2] Ainley V. Tajadura-Jiménez A. Fotopoulou A. Tsakiris M. (2012). Looking into myself: the effect of self-focused attention on interoceptive sensitivity. Psychophysiology 49:1504. doi: 10.1111/J.1469-8986.2012.01468.X 22978299 PMC3755258

[ref3] Al E. Iliopoulos F. Forschack N. Nierhaus T. Grund M. Motyka P. . (2020). Heart-brain interactions shape somatosensory perception and evoked potentials. PNAS 117, 10575–10584. doi: 10.1073/pnas.1915629117/-/DCSupplemental 32341167 PMC7229654

[ref4] Alleva J. M. Tylka T. L. (2021). Body functionality: a review of the literature. Body Image 36, 149–171. doi: 10.1016/J.BODYIM.2020.11.006, 33321273

[ref5] Apolone G. Mosconi P. (1998). The Italian SF-36 health survey: translation, validation and norming. J. Clin. Epidemiol. 51, 1025–1036. doi: 10.1016/S0895-4356(98)00094-8, 9817120

[ref6] Ardizzi M. Ferri F. (2018). Interoceptive influences on peripersonal space boundary. Cognition 177, 79–86. doi: 10.1016/j.cognition.2018.04.001, 29655026

[ref7] Azañón E. Mihaljevic K. Longo M. R. (2016). A three-dimensional spatial characterization of the crossed-hands deficit. Cognition 157, 289–295. doi: 10.1016/J.COGNITION.2016.09.007, 27697737

[ref8] Badoud D. Tsakiris M. (2017). From the body’s viscera to the body’s image: is there a link between interoception and body image concerns? Neurosci. Biobehav. Rev. 77, 237–246. doi: 10.1016/j.neubiorev.2017.03.017, 28377099

[ref9] Bailey K. A. Gammage K. L. van Ingen C. Ditor D. S. (2015). “It’s all about acceptance”: a qualitative study exploring a model of positive body image for people with spinal cord injury. Body Image 15, 24–34. doi: 10.1016/J.BODYIM.2015.04.010, 26002149

[ref10] Barrett L. F. (2017). The theory of constructed emotion: an active inference account of interoception and categorization. Soc. Cogn. Affect. Neurosci. 12, 1–23. doi: 10.1093/SCAN/NSW154, 27798257 PMC5390700

[ref11] Barsalou L. W. (2008). Grounded cognition. Annu. Rev. Psychol. 59, 617–645. doi: 10.1146/ANNUREV.PSYCH.59.103006.093639/CITE/REFWORKS 17705682

[ref12] Beard J. R. Officer A. De Carvalho I. A. Sadana R. Pot A. M. Michel J. P. . (2015). The world report on ageing and health: a policy framework for healthy ageing. Lancet 387, 2145–2154. doi: 10.1016/S0140-6736(15)00516-4, 26520231 PMC4848186

[ref13] Benton A. L. Sivan A. B. Hamsher K. Varney N. R. Spreen O. (1983). Contributions to neuropsychological assessment: Tests: 9. Finger localization complete test. USA: Oxford University Press.

[ref14] Binder M. (2015). Neural correlates of audiovisual temporal processing--comparison of temporal order and simultaneity judgments. Neuroscience 300, 432–447. doi: 10.1016/J.NEUROSCIENCE.2015.05.011, 25982561

[ref15] Brazier J. E. Harper R. Jones N. M. B. O’Cathain A. Thomas K. J. Usherwood T. . (1992). Validating the SF-36 health survey questionnaire: new outcome measure for primary care. BMJ (Clin. Res. Ed.). 305:160. doi: 10.1136/BMJ.305.6846.160 PMC1883187 1285753

[ref16] Brener J. Ring C. (2016). Towards a psychophysics of interoceptive processes: the measurement of heartbeat detection. Philos. Trans. R. Soc. Lond. Ser. B Biol. Sci. 371:20160015. doi: 10.1098/rstb.2016.0015, 28080972 PMC5062103

[ref17] Calì G. Ambrosini E. Picconi L. Mehling W. E. Committeri G. (2015). Investigating the relationship between interoceptive accuracy, interoceptive awareness, and emotional susceptibility. Front. Psychol. 6:1202. doi: 10.3389/fpsyg.2015.01202, 26379571 PMC4547010

[ref18] Canzoneri E. Magosso E. Serino A. (2012). Dynamic sounds capture the boundaries of peripersonal space representation in humans. PLoS One 7:e44306. doi: 10.1371/JOURNAL.PONE.0044306, 23028516 PMC3460958

[ref19] Carson N. Leach L. Murphy K. J. (2018). A re-examination of Montreal cognitive assessment (MoCA) cutoff scores. Int. J. Geriatr. Psychiatry 33, 379–388. doi: 10.1002/GPS.4756 28731508

[ref20] Carstensen L. L. Isaacowitz D. M. Charles S. T. (1999). Taking time seriously: a theory of socioemotional selectivity. Am. Psychol. 54, 165–181. doi: 10.1037/0003-066X.54.3.165, 10199217

[ref21] Carstensen L. L. Turan B. Scheibe S. Ram N. Ersner-Hershfield H. Samanez-Larkin G. R. . (2011). Emotional experience improves with age: evidence based on over 10 years of experience sampling. Psychol. Aging 26, 21–33. doi: 10.1037/A0021285, 20973600 PMC3332527

[ref22] Cesari M. De Carvalho I. A. Thiyagarajan J. A. Cooper C. Martin F. C. Reginster J. Y. . (2018). Evidence for the domains supporting the construct of intrinsic capacity. J. Gerontol. A Biol. Sci. Med. Sci. 73, 1653–1660. doi: 10.1093/GERONA/GLY011, 29408961

[ref23] Charles S. T. Carstensen L. L. (2010). Social and emotional aging. Annu. Rev. Psychol. 61:383. doi: 10.1146/ANNUREV.PSYCH.093008.100448, 19575618 PMC3950961

[ref24] Chepisheva M. K. (2023). Spatial orientation, postural control and the vestibular system in healthy elderly and Alzheimer’s dementia. PeerJ 11:e15040. doi: 10.7717/PEERJ.15040, 37151287 PMC10162042

[ref25] Christova P. S. Lewis S. M. Tagaris G. A. Uğurbil K. Georgopoulos A. P. (2008). A voxel-by-voxel parametric fMRI study of motor mental rotation: hemispheric specialization and gender differences in neural processing efficiency. Exp. Brain Res. 189, 79–90. doi: 10.1007/S00221-008-1405-X, 18478211

[ref26] Cohen C. I. Benyaminov R. Rahman M. Ngu D. Reinhardt M. (2023). Frailty: a multidimensional biopsychosocial syndrome. Med. Clin. North Am. 107, 183–197. doi: 10.1016/J.MCNA.2022.04.006, 36402498

[ref27] Costello M. C. Bloesch E. K. (2017). Are older adults less embodied? A review of age effects through the lens of embodied cognition. Front. Psychol. 8:239736. doi: 10.3389/FPSYG.2017.00267/PDF, 28289397 PMC5326803

[ref28] Covinsky K. E. Palmer R. M. Fortinsky R. H. Counsell S. R. Stewart A. L. Kresevic D. . (2003). Loss of independence in activities of daily living in older adults hospitalized with medical illnesses: increased vulnerability with age. J. Am. Geriatr. Soc. 51, 451–458. doi: 10.1046/J.1532-5415.2003.51152.X, 12657063

[ref29] Craig A. D. (2009). How do you feel — now? The anterior insula and human awareness. Nat. Rev. Neurosci. 10, 59–70. doi: 10.1038/nrn2555, 19096369

[ref30] Craig J. C. Rhodes R. P. Busey T. A. Diane K. P. Humes L. E. (2010). Aging and tactile temporal order. Atten. Percept. Psychophys. 72, 226–235. doi: 10.3758/APP.72.1.226, 20045891

[ref31] Critchley H. D. Garfinkel S. N. (2017). Interoception and emotion. Curr. Opin. Psychol. 17, 7–14. doi: 10.1016/J.COPSYC.2017.04.020 28950976

[ref32] Critchley H. D. Wiens S. Rotshtein P. Öhman A. Dolan R. J. (2004). Neural systems supporting interoceptive awareness. Nat. Neurosci. 7, 189–195. doi: 10.1038/nn1176 14730305

[ref33] Della Sala S. MacPherson S. E. Phillips L. H. Sacco L. Spinnler H. (2003). How many camels are there in Italy? Cognitive estimates standardised on the Italian population. Neurol. Sci. 24, 10–15. doi: 10.1007/S100720300015, 12754651

[ref34] Di Cosmo G. Costantini M. Salone A. Martinotti G. Di Iorio G. Di Giannantonio M. . (2018). Peripersonal space boundary in schizotypy and schizophrenia. Schizophr. Res. 197, 589–590. doi: 10.1016/j.schres.2017.12.003 29269210

[ref35] Di Cosmo G. Costantini M. Spadone S. Pizzella V. Della Penna S. Marzetti L. . (2021). Phase-coupling of neural oscillations contributes to individual differences in peripersonal space. Neuropsychologia 156:107823. doi: 10.1016/j.neuropsychologia.2021.107823, 33705822

[ref36] di Pellegrino G. Làdavas E. (2015). Peripersonal space in the brain. Neuropsychologia 66, 126–133. doi: 10.1016/J.NEUROPSYCHOLOGIA.2014.11.011 25448862

[ref37] Fazekas C. Avian A. Noehrer R. Matzer F. Vajda C. Hannich H. . (2022). Interoceptive awareness and self-regulation contribute to psychosomatic competence as measured by a new inventory. Wien. Klin. Wochenschr. 134, 581–592. doi: 10.1007/S00508-020-01670-5, 32430611 PMC9418284

[ref38] Feldman M. J. Bliss-Moreau E. Lindquist K. A. (2024). The neurobiology of interoception and affect. Trends Cogn. Sci. 28, 643–661. doi: 10.1016/J.TICS.2024.01.009, 38395706 PMC11222051

[ref39] Feller K. J. Peterka R. J. Horak F. B. (2019). Sensory re-weighting for postural control in Parkinson’s disease. Front. Hum. Neurosci. 13:126. doi: 10.3389/FNHUM.2019.00126, 31057379 PMC6478764

[ref40] Ferri F. Ambrosini E. Costantini M. (2016). Spatiotemporal processing of somatosensory stimuli in schizotypy. Sci. Rep. 6:38735. doi: 10.1038/srep38735, 27934937 PMC5146666

[ref41] Ferri F. Costantini M. Huang Z. Perrucci M. G. Ferretti A. Romani G. L. . (2015). Intertrial variability in the premotor cortex accounts for individual differences in peripersonal space. J. Neurosci. 35, 16328–16339. doi: 10.1523/JNEUROSCI.1696-15.2015, 26674860 PMC6605506

[ref42] Ferroni F. Ardizzi M. Magnani F. Ferri F. Langiulli N. Rastelli F. . (2022). Tool-use extends peripersonal space boundaries in schizophrenic patients. Schizophr. Bull. 48, 1085–1093. doi: 10.1093/schbul/sbac067, 35708490 PMC9434469

[ref43] Fittipaldi S. Abrevaya S. Fuente A. de la Pascariello G. O. Hesse E. Birba A. . 2020 A multidimensional and multi-feature framework for cardiac interoception NeuroImage 212:116677 doi: 10.1016/J.NEUROIMAGE.2020.116677 32101777 PMC7165068

[ref44] Foisy A. Kapoula Z. (2018). Plantar cutaneous afferents influence the perception of subjective visual vertical in quiet stance. Sci. Rep. 8:14939. doi: 10.1038/S41598-018-33268-3, 30297709 PMC6175839

[ref45] Folstein M. F. Folstein S. E. McHugh P. R. (1975). “Mini-mental state”: a practical method for grading the cognitive state of patients for the clinician. J. Psychiatr. Res. 12, 189–198. doi: 10.1016/0022-3956(75)90026-6, 1202204

[ref46] Frisén A. Holmqvist K. (2010). What characterizes early adolescents with a positive body image? A qualitative investigation of Swedish girls and boys. Body Image 7, 205–212. doi: 10.1016/J.BODYIM.2010.04.001, 20554256

[ref47] Füstös J. Gramann K. Herbert B. M. Pollatos O. (2013). On the embodiment of emotion regulation: interoceptive awareness facilitates reappraisal. Soc. Cogn. Affect. Neurosci. 8, 911–917. doi: 10.1093/SCAN/NSS089, 22933520 PMC3831556

[ref48] Gallese V. Sinigaglia C. (2011). What is so special about embodied simulation? Trends Cogn. Sci. 15, 512–519. doi: 10.1016/j.tics.2011.09.003, 21983148

[ref49] García-Cordero I. Esteves S. Mikulan E. P. Hesse E. Baglivo F. H. Silva W. . (2017). Attention, in and out: scalp-level and intracranial EEG correlates of interoception and exteroception. Front. Neurosci. 11:411. doi: 10.3389/fnins.2017.00411 28769749 PMC5515904

[ref50] Garfinkel S. N. Seth A. K. Barrett A. B. Suzuki K. Critchley H. D. (2015). Knowing your own heart: distinguishing interoceptive accuracy from interoceptive awareness. Biol. Psychol. 104, 65–74. doi: 10.1016/J.BIOPSYCHO.2014.11.004, 25451381

[ref51] Gattario K. H. Frisén A. (2019). From negative to positive body image: men’s and women’s journeys from early adolescence to emerging adulthood. Body Image 28, 53–65. doi: 10.1016/J.BODYIM.2018.12.002, 30583277

[ref52] Grund M. Al E. Pabst M. Dabbagh A. Stephani T. Nierhaus T. . (2022). Respiration, heartbeat, and conscious tactile perception. J. Neurosci. 42, 643–656. doi: 10.1523/JNEUROSCI.0592-21.2021, 34853084 PMC8805629

[ref53] Guralnik J. M. Fried L. P. Salive M. E. (1996). Disability as a public health outcome in the aging population. Annu. Rev. Public Health 17, 25–46. doi: 10.1146/ANNUREV.PU.17.050196.000325, 8724214

[ref54] Han J. Park S. Jung S. Choi Y. Song H. (2015). Comparisons of changes in the two-point discrimination test following muscle fatigue in healthy adults. J. Phys. Ther. Sci. 27, 551–554. doi: 10.1589/JPTS.27.551, 25931678 PMC4395662

[ref55] Harvie D. S. Edmond-Hank G. Smith A. D. (2018). Tactile acuity is reduced in people with chronic neck pain. Musculoskeletal Sci. Pract. 33, 61–66. doi: 10.1016/j.msksp.2017.11.009, 29180111

[ref56] Hietanen J. K. Glerean E. Hari R. Nummenmaa L. (2016). Bodily maps of emotions across child development. Dev. Sci. 19, 1111–1118. doi: 10.1111/desc.12389, 26898716

[ref57] Jekel K. Damian M. Wattmo C. Hausner L. Bullock R. Connelly P. J. . (2015). Mild cognitive impairment and deficits in instrumental activities of daily living: a systematic review. Alzheimer's Res. Ther. 7:17. doi: 10.1186/S13195-015-0099-0, 25815063 PMC4374414

[ref001] International Monetary Fund, 2012. International Monetary Fund Annual Report 2012: Working Together To Support Global Recovery. (USA: International Monetary Fund) (2012). doi: 10.5089/9781616354152.011

[ref58] Kekäläinen T. Luchetti M. Sutin A. Terracciano A. (2023). Functional capacity and difficulties in activities of daily living from a cross-national perspective. J. Aging Health 35, 356–369. doi: 10.1177/08982643221128929, 36245236 PMC10104963

[ref59] Khalsa S. S. Rudrauf D. Tranel D. (2009). Interoceptive awareness declines with age. Psychophysiology 46, 1130–1136. doi: 10.1111/J.1469-8986.2009.00859.X, 19602175 PMC2865139

[ref60] Kim J. Park H.-D. Woon Kim K. Woo Shin D. Lim S. Kwon H. . (2025). Sad faces increase the heartbeat-associated interoceptive information flow within the salience network: a MEG study. Sci. Rep. 23:430. doi: 10.1038/s41598-018-36498-7 PMC6344475 30674995

[ref61] Koch A. Pollatos O. Mehling W. E. Sänger J. Dunn B. (2014). Interoceptive sensitivity, body weight and eating behavior in children: a prospective study. Front. Psychol. 5:1003. doi: 10.3389/fpsyg.2014.01003 25250006 PMC4158976

[ref62] Kostaki M. Vatakis A. (2018). “Temporal order and synchrony judgments: a primer for students” in Timing and time perception: Procedures, measures, and applications (Leiden, The Netherlands: BRILL Press), 233–262. doi: 10.1163/9789004280205_012

[ref63] Kuehn E. Perez-Lopez M. B. Diersch N. Döhler J. Wolbers T. Riemer M. (2018). Embodiment in the aging mind. Neurosci. Biobehav. Rev. 86, 207–225. doi: 10.1016/J.NEUBIOREV.2017.11.016, 29175306

[ref64] Kunzmann U. Little T. D. Smith J. (2000). Is age-related stability of subjective well-being a paradox? Cross-sectional and longitudional evidence from the Berlin aging study. Psychol. Aging 15, 511–526. doi: 10.1037//0882-7974.15.3.511, 11014714

[ref65] Laasonen M. Service E. Virsu V. (2001). Temporal order and processing acuity of visual, auditory, and tactile perception in developmentally dyslexic young adults. Cogn. Affect. Behav. Neurosci. 1, 394–410. doi: 10.3758/CABN.1.4.394 12467091

[ref66] Laughter S. Mitchell V. Nguyen N. Kim C. (2020). Interrelationship between sensory modulation, altered interoceptive awareness, and anxiety and impacts on quality of life.

[ref67] Liu M. Dudarev V. de Brouwer A. J. Enns J. T. (2025). Perceiving exertion in others: from interoception to exteroception. Psychon. Bull. Rev. 32, 2696–2718. doi: 10.3758/S13423-025-02753-Y, 40775588

[ref68] Löffler A. Beier F. Bekrater-Bodmann R. Hausner L. Desch S. Silvoni S. . (2024). Reduced tactile sensitivity is associated with mild cognitive impairment. EBioMedicine 99:104896. doi: 10.1016/J.EBIOM.2023.104896, 38041920 PMC10711381

[ref69] Longo M. R. (2016). Types of body representation. In: eds. Coello Y. Fischer M. H., Foundations of Embodied Cognition, Volume 1: Perceptual and Emotional Embodiment. London: Routledge. 117–134.

[ref70] MacCormack J. K. Feldman M. J. Bonar A. S. Lindquist K. A. (2023). “Aging bodies, brains, and emotions” in Emotion communication by the aging face and body, 54–82.

[ref9004] MacCormack J. K. Feldman M. J. Bonar A. S. Lindquist K. A. (2023). Aging bodies, brains, and emotions: The Physiological Hypothesis of Emotional Aging. In: Hess U Jr. Adams RB Kleck RE, eds. Emotion Communication by the Aging Face and Body: A Multidisciplinary View. Studies in Emotion and Social Interaction. Cambridge University Press. 54–82.

[ref71] MacPherson S. E. Wagner G. P. Murphy P. Bozzali M. Cipolotti L. Shallice T. (2014). Bringing the cognitive estimation task into the 21st century: normative data on two new parallel forms. PLoS One 9:e92554. doi: 10.1371/JOURNAL.PONE.0092554, 24671170 PMC3966793

[ref72] Magni E. Binetti G. Bianchetti A. Rozzini R. Trabucchi M. (1996). Mini-mental state examination: a normative study in Italian elderly population. Eur. J. Neurol. 3, 198–202. doi: 10.1111/J.1468-1331.1996.TB00423.X, 21284770

[ref73] Mahoney J. R. Cotton K. Verghese J. Newman A. (2019). Multisensory integration predicts balance and falls in older adults. J. Gerontol. A Biol. Sci. Med. Sci. 74, 1429–1435. doi: 10.1093/gerona/gly245, 30357320 PMC6696711

[ref74] Mahoney J. R. Verghese J. (2019). Using the race model inequality to quantify behavioral multisensory integration effects. J. Vis. Exp. 2019:10.3791/59575. doi: 10.3791/59575, 31132070 PMC9425836

[ref75] Marcora S. M. Staiano W. Manning V. (2009). Mental fatigue impairs physical performance in humans. J. Appl. Physiol. 106, 857–864. doi: 10.1152/JAPPLPHYSIOL.91324.2008, 19131473

[ref76] Masson C. Van Der Westhuizen D. Noel J.-P. Prevost A. Jack Van Honk, ·, Fotopoulou A. Mark Solms Serino A. (2021). Testosterone administration in women increases the size of their peripersonal space. 239, 1639–1649. doi: 10.1007/s00221-021-06080-1 33770219

[ref77] Mattioni L. Sestieri C. Perrucci M. G. Spada M. M. Ferri F. (2024). The role of interoceptive awareness in shaping the relationship between desire thinking and cigarette consumption. Int. J. Psychophysiol. 201:112369. doi: 10.1016/J.IJPSYCHO.2024.112369, 38768660

[ref78] Mayer E. A. Naliboff B. D. Craig A. D. B. (2006). Neuroimaging of the brain-gut Axis: from basic understanding to treatment of functional GI disorders. Gastroenterology 131, 1925–1942. doi: 10.1053/J.GASTRO.2006.10.026, 17188960

[ref79] McFarland R. A. (1975). Heart rate perception and heart rate control. Psychophysiology 12, 402–405. doi: 10.1111/j.1469-8986.1975.tb00011.x 1162006

[ref80] McIntyre S. Nagi S. S. McGlone F. Olausson H. (2021). The effects of ageing on tactile function in humans. Neuroscience 464, 53–58. doi: 10.1016/J.NEUROSCIENCE.2021.02.015, 33607227

[ref81] McMorris T. (2020). Cognitive fatigue effects on physical performance: the role of interoception. Sports Med. 50, 1703–1708. doi: 10.1007/S40279-020-01320-W/FIGURES/1 32661840

[ref82] Mehling W. E. Price C. Daubenmier J. J. Acree M. Bartmess E. (2012). The multidimensional assessment of interoceptive awareness (MAIA). PLoS One 7:48230. doi: 10.1371/journal.pone.0048230, 23133619 PMC3486814

[ref83] Mendes W. B. (2010). Weakened links between mind and body in older age: the case for maturational dualism in the experience of emotion. Emotion Rev. 2, 240–244. doi: 10.1177/1754073910364149

[ref84] Mibu A. Kan S. Nishigami T. Fujino Y. Shibata M. (2020). Performing the hand laterality judgement task does not necessarily require motor imagery. Sci. Rep. 10: 5155. doi: 10.1038/s41598-020-61937-9, 32198401 PMC7083854

[ref9002] Miller J Ulrich R. On the analysis of psychometric functions: the Spearman-Kärber method. Percept Psychophys. (2001). 63:1399–420. doi: 10.3758/bf03194551 11800465

[ref85] Monti A. Porciello G. Panasiti M. S. Aglioti S. M. (2021). The inside of me: interoceptive constraints on the concept of self in neuroscience and clinical psychology. Psychol. Res. 86, 2468–2477. doi: 10.1007/S00426-021-01477-7, 34050431 PMC9674731

[ref86] Mueller S. M. Grunwald M. (2023). “Perceptual dimensions of the haptic system” in Human touch in healthcare. Berlin, Heidelberg: Springer. 1–41. doi: 10.1007/978-3-662-67860-2_1

[ref87] Murman D. L. (2015). The impact of age on cognition. Semin. Hear. 36, 111–121. doi: 10.1055/s-0035-1555115, 27516712 PMC4906299

[ref88] Murphy J. Geary H. Millgate E. Catmur C. Bird G. (2018). Direct and indirect effects of age on interoceptive accuracy and awareness across the adult lifespan. Psychon. Bull. Rev. 25, 1193–1202. doi: 10.3758/S13423-017-1339-Z, 28685271 PMC5990557

[ref89] Myachykov A. Scheepers C. Fischer M. H. Kessler K. (2014). TEST: a tropic, embodied, and situated theory of cognition. Top. Cogn. Sci. 6, 442–460. doi: 10.1111/tops.12024, 23616259

[ref90] Naor-Ziv R. King R. Glicksohn J. (2020). Rank-order of body shapes reveals internal hierarchy of body image. J. Person-Oriented Res. 6, 28–38. doi: 10.17505/jpor.2020.22044, 33569150 PMC7842620

[ref91] Nasreddine Z. S. Phillips N. A. Bédirian V. Charbonneau S. Whitehead V. Collin I. . (2005). The Montreal cognitive assessment, MoCA: a brief screening tool for mild cognitive impairment. J. Am. Geriatr. Soc. 53, 695–699. doi: 10.1111/J.1532-5415.2005.53221.X, 15817019

[ref92] Nelesen R. Dar Y. Thomas K. Dimsdale J. E. (2008). The relationship between fatigue and cardiac functioning. Arch. Intern. Med. 168:943. doi: 10.1001/ARCHINTE.168.9.943, 18474758 PMC2633298

[ref93] Nord C. L. Garfinkel S. N. (2022). Interoceptive pathways to understand and treat mental health conditions. Trends Cogn. Sci. 26, 499–513. doi: 10.1016/J.TICS.2022.03.004/ASSET/135C9456-E7AC-431F-809C-B9C28D345409/MAIN.ASSETS/GR2.JPG, 35466044

[ref94] Nummenmaa L. Glerean E. Hari R. Hietanen J. K. (2014). Bodily maps of emotions. Proc. Natl. Acad. Sci. USA 111, 646–651. doi: 10.1073/pnas.1321664111, 24379370 PMC3896150

[ref95] O’Carroll R. Egan V. MacKenzie D. M. (1994). Assessing cognitive estimation. Br. J. Clin. Psychol. 33, 193–197. doi: 10.1111/J.2044-8260.1994.TB01110.X 8038735

[ref96] Pan J. Tompkins W. J. (1985). A real-time QRS detection algorithm. I.E.E.E. Trans. Biomed. Eng. 32, 230–236. doi: 10.1109/TBME.1985.325532, 3997178

[ref97] Pasciucco M. R. Nunziata S. Iuliano S. Perrucci M. G. Costantini M. Ruggiero G. . (2025a). Impact of interoception and multisensory integration on functional and physical activities in aging. Cortex 194, 107–120. doi: 10.1016/j.cortex.2025.11.010, 41389593

[ref98] Pasciucco M. R. Perrucci M. G. Croce P. Kalckert A. Costantini M. Ferri F. (2025b). Predictive role of exteroceptive and interoceptive bodily dimensions to schizotypal personality traits. Sci. Rep. 15:7909. doi: 10.1038/S41598-025-89951-9, 40050671 PMC11885456

[ref99] Peterka R. J. (2002). Sensorimotor integration in human postural control. J. Neurophysiol. 88, 1097–1118. doi: 10.1152/JN.2002.88.3.1097, 12205132

[ref100] Pfeifer G. Cawkwell S. (2025). Interoceptive ageing and the impact on psychophysiological processes: a systematic review. Int. J. Psychophysiol. 207:112483. doi: 10.1016/J.IJPSYCHO.2024.112483, 39657288

[ref101] Poli A. Maremmani A. G. I. Chiorri C. Mazzoni G. P. Orrù G. Kolacz J. . (2021). Item reduction, psychometric and biometric properties of the Italian version of the body perception questionnaire—short form (BPQ-sf): the bpq-22. Int. J. Environ. Res. Public Health 18:3835. doi: 10.3390/ijerph18073835, 33917552 PMC8038843

[ref102] Porges S. (1993). Body perception questionnaire: Laboratory of Developmental Assessment, University of Maryland, 10.

[ref103] Price C. J. Hooven C. (2018). Interoceptive awareness skills for emotion regulation: theory and approach of mindful awareness in body-oriented therapy (MABT). Front. Psychol. 9:798. doi: 10.3389/FPSYG.2018.00798, 29892247 PMC5985305

[ref104] Qi M. Shen X. Zeng Y. Lin X. Suliman M. Li P. (2025). Interoception and mental health in middle-aged and elderly adults: a systematic review and meta-analysis. Neurosci. Biobehav. Rev. 172:106104. doi: 10.1016/J.NEUBIOREV.2025.106104, 40081440

[ref105] Rabellino D. Frewen P. A. McKinnon M. C. Lanius R. A. (2020). Peripersonal space and bodily self-consciousness: implications for psychological trauma-related disorders. Front. Neurosci. 14:586605. doi: 10.3389/fnins.2020.586605, 33362457 PMC7758430

[ref106] Raichle M. E. MacLeod A. M. Snyder A. Z. Powers W. J. Gusnard D. A. Shulman G. L. (2001). A default mode of brain function. Proc. Natl. Acad. Sci. USA 98, 676–682. doi: 10.1073/PNAS.98.2.676 11209064 PMC14647

[ref107] Raimo S. Di Vita A. Boccia M. Iona T. Cropano M. Gaita M. . (2021). The body across the lifespan: on the relation between interoceptive sensibility and high-order body representations. Brain Sci. 11:493. doi: 10.3390/BRAINSCI11040493/S1 33924634 PMC8070580

[ref108] Ruggiero G. Frassinetti F. Coello Y. Rapuano M. di Cola A. S. Iachini T. (2017). The effect of facial expressions on peripersonal and interpersonal spaces. Psychol. Res. 81, 1232–1240. doi: 10.1007/S00426-016-0806-X/METRICS, 27785567

[ref109] Saltafossi M. Zaccaro A. Kluger D. S. Perrucci M. G. Ferri F. Costantini M. (2025). Respiration facilitates behaviour during multisensory integration. bioRxiv:2025.01.10.632352. doi: 10.1101/2025.01.10.632352 PMC12464358 40999648

[ref110] Saltafossi M. Zaccaro A. Perrucci M. G. Ferri F. Costantini M. (2023). The impact of cardiac phases on multisensory integration. Biol. Psychol. 182:108642. doi: 10.1016/j.biopsycho.2023.108642, 37467844

[ref111] Sardella A. Lenzo V. Basile G. Martino G. Quattropani M. C. (2023). Emotion regulation strategies and difficulties in older adults: a systematic review. Clin. Gerontol. 46, 280–301. doi: 10.1080/07317115.2022.2128706 36163629

[ref112] Sarko D. K. Nidiffer A. R. Powers A. R. Ghose D. Hillock-Dunn A. Fister M. C. . (2012). Spatial and temporal features of multisensory processes: bridging animal and human studies. In eds. Murray MM Wallace MT. The neural bases of multisensory processes, Boca Raton (FL): CRC Press/Taylor & Francis. 191–215. Available online at: https://www.ncbi.nlm.nih.gov/books/NBK92831/22593860

[ref113] Schandry R. (1981). Heart beat perception and emotional experience. Psychophysiology 18, 483–488. doi: 10.1111/j.1469-8986.1981.tb02486.x, 7267933

[ref114] Schmitt C. M. Schoen S. (2022). Interoception: a multi-sensory Foundation of Participation in daily life. Front. Neurosci. 16:875200. doi: 10.3389/fnins.2022.875200, 35757546 PMC9220286

[ref115] Serino A. (2019). Peripersonal space (PPS) as a multisensory interface between the individual and the environment, defining the space of the self. Neurosci. Biobehav. Rev. 99, 138–159. doi: 10.1016/J.NEUBIOREV.2019.01.016, 30685486

[ref116] Seth A. K. (2013). Interoceptive inference, emotion, and the embodied self. Trends Cogn. Sci. 17, 565–573. doi: 10.1016/J.TICS.2013.09.007/ASSET/47B8FDBF-502F-4D09-B189-D9BFDF3B553F/MAIN.ASSETS/GR3.JPG, 24126130

[ref117] Shadmehr R. Krakauer J. W. (2008). A computational neuroanatomy for motor control. Exp. Brain Res. 185, 359–381. doi: 10.1007/S00221-008-1280-5 18251019 PMC2553854

[ref118] Spadone S. Perrucci M. G. Di Cosmo G. Costantini M. Della Penna S. Ferri F. (2021). Frontal and parietal background connectivity and their dynamic changes account for individual differences in the multisensory representation of peripersonal space. Sci. Rep. 11:20533. doi: 10.1038/s41598-021-00048-5, 34654814 PMC8520015

[ref119] Stenner P. McFarquhar T. Bowling A. (2011). Older people and “active ageing”: subjective aspects of ageing actively. J. Health Psychol. 16, 467–477. doi: 10.1177/1359105310384298, 21224334

[ref120] Stevens J. C. Cruz L. A. (1996). Spatial acuity of touch: ubiquitous decline with aging revealed by repeated threshold testing. Somatosens. Mot. Res. 13, 1–10. doi: 10.3109/08990229609028907, 8725644

[ref121] Stuck A. E. Walthert J. M. Nikolaus T. Büla C. J. Hohmann C. Beck J. C. (1999). Risk factors for functional status decline in community-living elderly people: a systematic literature review. Soc. Sci. Med. 48, 445–469. doi: 10.1016/S0277-9536(98)00370-0, 10075171

[ref122] Tarvainen M. P. Niskanen J. P. Lipponen J. A. Ranta-aho P. O. Karjalainen P. A. (2014). Kubios HRV – heart rate variability analysis software. Comput. Methods Prog. Biomed. 113, 210–220. doi: 10.1016/J.CMPB.2013.07.024, 24054542

[ref123] Thayer J. F. Åhs F. Fredrikson M. Sollers J. J. Wager T. D. (2012). A meta-analysis of heart rate variability and neuroimaging studies: implications for heart rate variability as a marker of stress and health. Neurosci. Biobehav. Rev. 36, 747–756. doi: 10.1016/J.NEUBIOREV.2011.11.009, 22178086

[ref124] Todd J. Aspell J. E. Barron D. Swami V. (2019). An exploration of the associations between facets of interoceptive awareness and body image in adolescents. Body Image 31, 171–180. doi: 10.1016/J.BODYIM.2019.10.004, 31654981

[ref125] Ulus G. Aisenberg-Shafran D. (2022). Interoception in old age. Brain Sci. 12:1398. doi: 10.3390/brainsci12101398, 36291331 PMC9599927

[ref126] van Oostrom S. H. van der A D. L. Liset Rietman M. Susan Picavet H. J. Lette M. Monique Verschuren W. M. . (2017). A four-domain approach of frailty explored in the Doetinchem cohort study. BMC Geriatr. 17:196. doi: 10.1186/s12877-017-0595-0 28854882 PMC5577839

[ref127] Vlachou M. E. Legros J. Sellin C. Paleressompoulle D. Massi F. Simoneau M. . (2025). Tactile contribution extends beyond exteroception during spatially guided finger movements. Sci. Rep. 15:14959-. doi: 10.1038/s41598-025-99503-w, 40301588 PMC12041493

[ref128] Wada M. Suzuki M. Takaki A. Miyao M. Spence C. Kansaku K. (2014). Spatio-temporal processing of tactile stimuli in autistic children. Sci. Rep. 4:5985. doi: 10.1038/srep05985 25100146 PMC4124471

[ref129] Wallman-Jones A. Perakakis P. Tsakiris M. Schmidt M. (2021). Physical activity and interoceptive processing: theoretical considerations for future research. Int. J. Psychophysiol. 166, 38–49. doi: 10.1016/J.IJPSYCHO.2021.05.002, 33965423

[ref130] Walsh V. (2003). A theory of magnitude: common cortical metrics of time, space and quantity. Trends Cogn. Sci. 7, 483–488. doi: 10.1016/J.TICS.2003.09.002, 14585444

[ref131] Wang W. Liu Y. Ji D. Xie K. Yang Y. Zhu X. . (2024). The association between functional disability and depressive symptoms among older adults: findings from the China health and retirement longitudinal study (CHARLS). J. Affect. Disord. 351, 518–526. doi: 10.1016/J.JAD.2024.01.256, 38307133

[ref132] Weber E. H. (1996). On the tactile senses. E. H. Weber on the tactile senses (2nd ed.) eds. H. E. Ross and D. J. Murray. Erlbaum (Uk): Taylor & Francis, Publ.

[ref133] Weiss S. Sack M. Henningsen P. Pollatos O. (2014). On the interaction of self-regulation, interoception and pain perception. Psychopathology 47, 377–382. doi: 10.1159/000365107, 25342240

[ref134] Wilson R. E. Latner J. D. Hayashi K. (2013). More than just body weight: the role of body image in psychological and physical functioning. Body Image 10, 644–647. doi: 10.1016/J.BODYIM.2013.04.007, 23726517

[ref1001] World Health Organization. (2002). The World Health Report 2002: Reducing Risks, Promoting Healthy Life. Geneva: World Health Organization.

[ref1002] World Health Organization. (2020). World Health Statistics 2020: Monitoring health for the SDGs. Geneva: World Health Organization.

[ref135] Wolpert D. M. Diedrichsen J. Flanagan J. R. (2011). Principles of sensorimotor learning. Nat. Rev. Neurosci. 12, 739–751. doi: 10.1038/NRN3112, 22033537

[ref136] Wróblewska Z. Chmielewski J. P. Florek-Łuszczki M. Nowak-Starz G. Wojciechowska M. Wróblewska I. M. (2023). Assessment of functional capacity of the elderly. Ann. Agric. Environ. Med. 30, 156–163. doi: 10.26444/aaem/161775, 36999869

[ref137] Yamamoto S. Yamamoto S. (2001). Reversal of subjective temporal order due to arm crossing. Nat Neurosci. 4:759–65. doi: 10.1038/89559 11426234

[ref138] Yau J. M. DeAngelis G. C. Angelaki D. E. (2015). Dissecting neural circuits for multisensory integration and crossmodal processing. Philos. Trans. R. Soc. Lond. Ser. B Biol. Sci. 370:20140203. doi: 10.1098/RSTB.2014.0203, 26240418 PMC4528815

[ref139] Yoris A. García A. M. Traiber L. Santamaría-García H. Martorell M. Alifano F. . (2017). The inner world of overactive monitoring: neural markers of interoception in obsessive-compulsive disorder. Psychol. Med. 47, 1957–1970. doi: 10.1017/S0033291717000368, 28374658

[ref140] Zaccaro A. Perrucci M. G. Parrotta E. Costantini M. Ferri F. (2022). Brain-heart interactions are modulated across the respiratory cycle via interoceptive attention. NeuroImage 262:119548. doi: 10.1016/J.NEUROIMAGE.2022.119548, 35964864

[ref141] Zamariola G. Frost N. Van Oost A. Corneille O. Luminet O. (2019). Relationship between interoception and emotion regulation: new evidence from mixed methods. J. Affect. Disord. 246, 480–485. doi: 10.1016/J.JAD.2018.12.101, 30599372

