# Supplementary material for: Interoceptive predictors of daily functioning in aging and their interaction with exteroceptive bodily representations
Source: Front Psychol. 2026 Feb 6;17:1689759. doi: 10.3389/fpsyg.2026.1689759 (PMC12920490; doi:10.3389/fpsyg.2026.1689759)
Supplement: Supplementary file 1 [file Supplementary_file_1.docx]

**Supplementary material**

**Table S1 Summary of participants’ demographic characteristics, cognitive screening scores (MMSE, MoCA, CET), and scores for all interoceptive, exteroceptive, and multisensory variables included in the study.**

|  | **Young early adulthood**  **N=25** | | **Middle adulthood**  **N= 13** | | **Old age**  **N=22** | |
| --- | --- | --- | --- | --- | --- | --- |
|  | **M=13** | **F=12** | **M=2** | **F=11** | **M=5** | **F=17** |
|  | **MEAN** | **SD** | **MEAN** | **SD** | **MEAN** | **SD** |
| **Age** | 30,12 | 5,98 | 50,62 | 6,44 | 68,36 | 6,69 |
| **SF-36** Short Form Health Survey | | | | | | |
| **Scales** | **MEAN** | **SD** | **MEAN** | **SD** | **MEAN** | **SD** |
| **Physical Functioning** | 98,00 | 4,56 | 91,15 | 13,56 | 83,64 | 13,02 |
| **Role Limitations due to physical health problems** | 84,00 | 30,52 | 73,08 | 34,55 | 80,68 | 33,57 |
| **Role Limitations due to personal/emotional problems** | 66,67 | 43,03 | 61,54 | 40,47 | 87,88 | 30,07 |
| **Energy/Fatigue** | 54,20 | 13,59 | 56,54 | 21,54 | 61,14 | 17,52 |
| **Emotional Well-Being** | 64,80 | 18,48 | 65,54 | 19,63 | 68,55 | 19,37 |
| **Social Functioning** | 72,50 | 25,52 | 65,38 | 17,79 | 78,41 | 19,74 |
| **Bodily Pain** | 84,00 | 20,05 | 70,58 | 20,90 | 78,30 | 16,64 |
| **General Health** | 70,40 | 21,93 | 65,38 | 17,85 | 60,68 | 14,00 |
| Cognitive screening | | | | | | |
| **Questionnaire** | **MEAN** | **SD** | **MEAN** | **SD** | **MEAN** | **SD** |
| **MMSE** Mini-Mental State Examination | 26,70 | 0,63 | 27,26 | 0,73 | 27,68 | 1,20 |
| **MoCa** Montereal Cognitive Assessment | 28,20 | 1,26 | 26,85 | 0,99 | 27,27 | 1,35 |
| **CET** Cognitive Estimation Test | 14,64 | 3,08 | 13,69 | 2,25 | 13,23 | 3,41 |
| Physiological measures | | | | | | |
| **Measures** | **MEAN** | **SD** | **MEAN** | **SD** | **MEAN** | **SD** |
| **BreathFrequency** | 14,37 | 3,80 | 14,76 | 2,05 | 17,15 | 4,04 |
| **Mean HR** | 73,58 | 8,04 | 72,99 | 9,12 | 66,65 | 8,66 |
| **HF** High Frequency | 5,77 | 1,04 | 4,88 | 0,86 | 4,38 | 1,16 |
| **LF/HF** Ratio between low frequency and high frequency | 0,64 | 0,19 | 0,42 | 0,21 | 0,38 | 0,22 |
| **MAIA** Multidimensional Assessment of Interoceptive Awareness | | | | | | |
| **Scales** | **MEAN** | **SD** | **MEAN** | **SD** | **MEAN** | **SD** |
| **M1** Noticing scale | 3,18 | 0,93 | 1,98 | 1,14 | 2,47 | 1,39 |
| **M2** Not-Distracting scale | 2,36 | 0,62 | 2,85 | 0,88 | 1,79 | 0,91 |
| **M3** Not-Worrying scale | 2,53 | 1,04 | 2,56 | 1,02 | 2,33 | 0,86 |
| **M4** Attention Regulation scale | 2,99 | 1,02 | 2,57 | 0,90 | 2,94 | 1,08 |
| **M5** Emotional Awareness scale | 3,42 | 0,94 | 3,25 | 0,90 | 3,43 | 1,14 |
| **M6** Self-Regulation scale | 2,61 | 1,13 | 2,60 | 1,11 | 2,91 | 1,10 |
| **M7** Body Listening scale | 2,65 | 1,10 | 2,44 | 1,44 | 2,70 | 1,50 |
| **M8** Trusting scale | 3,19 | 0,99 | 3,46 | 1,15 | 3,92 | 1,14 |
| **BPQ** Body Perception Questionnaire | | | | | | |
| **Scales** | **MEAN** | **SD** | **MEAN** | **SD** | **MEAN** | **SD** |
| **BPQ_BOA** Body Awareness | 16,32 | 3,69 | 13,38 | 4,35 | 12,82 | 3,25 |
| **BPQ_SUP** Supradiaphragmatic Reactivity | 10,60 | 2,92 | 10,23 | 3,00 | 11,00 | 2,41 |
| **BPQ_BOA/SUB** Body Awareness/Subdiaphragmatic reactivity | 15,44 | 2,83 | 14,15 | 4,16 | 12,82 | 2,81 |
| Interoceptive Tasks | | | | | | |
| **Variables** | **MEAN** | **SD** | **MEAN** | **SD** | **MEAN** | **SD** |
| **Acc-d** Accuracy in heartbeat detection task | 0,77 | 0,16 | 0,82 | 0,13 | 0,70 | 0,21 |
| **Con-d** Confidence in heartbeat detection task | 5,17 | 1,63 | 6,23 | 2,01 | 6,07 | 1,85 |
| **Aw-d** Awareness in heartbeat detection task | 0,73 | 0,22 | 0,82 | 0,18 | 0,70 | 0,26 |
| **Acc-c** Accuracy in heartbeat counting task | 0,76 | 0,18 | 0,78 | 0,11 | 0,66 | 0,20 |
| **Con-c** Confidence in heartbeat counting task | 6,57 | 1,21 | 6,65 | 1,22 | 6,99 | 1,24 |
| **Aw-c** Awareness in heartbeat counting task | 0,69 | 0,18 | 0,74 | 0,23 | 0,69 | 0,16 |
| **LJT** Laterality Judgment Task | | | | | | |
| **Variables** | **MEAN** | **SD** | **MEAN** | **SD** | **MEAN** | **SD** |
| **MRE-rh** Mental Rotation Efficiency – right hand | 230,17 | 105,00 | 246,76 | 119,55 | 270,90 | 174,90 |
| **MRE-lh** Mental Rotation Efficiency – left hand | 223,28 | 132,15 | 224,25 | 141,00 | 234,11 | 116,18 |
| **PPS** Peripersonal Space Task | | | | | | |
| **Variable** | **MEAN** | **SD** | **MEAN** | **SD** | **MEAN** | **SD** |
| **PSE-pps** Point of Subjective Equality | 1361,38 | 515,74 | 1486,01 | 417,29 | 1507,82 | 561,59 |
| **SJ** Simultaneity Judgment Task | | | | | | |
| **Variable** | **MEAN** | **SD** | **MEAN** | **SD** | **MEAN** | **SD** |
| **JND-sj** Just Noticeable Difference | 109,41 | 34,19 | 186,04 | 109,84 | -3285,65 | 15495,79 |
| **TOJ** Temporal Order Judgment Task | | | | | | |
| **Variables** | **MEAN** | **SD** | **MEAN** | **SD** | **MEAN** | **SD** |
| **JND-tojun** Just Noticeable Difference - Uncross condition | 54,65 | 27,39 | 111,88 | 92,86 | 150,02 | 207,32 |
| **JND-tojcr** Just Noticeable Difference – Cross condition | 64,17 | 38,34 | 165,08 | 280,25 | 134,79 | 116,85 |
| **SC** Sum of Confusion | 70,63 | 88,84 | 72,94 | 111,50 | 115,89 | 134,24 |
| **PFRS** Photographic Figure Rating Scale | | | | | | |
| **Variable** | **MEAN** | **SD** | **MEAN** | **SD** | **MEAN** | **SD** |
| **ΔAR** discrepancies between A and the participants BMI | 1,58 | 3,84 | 2,30 | 4,07 | 3,24 | 4,27 |
| **FLT** Finger Localization Task | | | | | | |
| **Variable** | **MEAN** | **SD** | **MEAN** | **SD** | **MEAN** | **SD** |
| **HIT** Correct response | 0,97 | 0,08 | 0,98 | 0,04 | 0,93 | 0,10 |
| **2PD** Two Point Discrimination Task | | | | | | |
| **Variables** | **MEAN** | **SD** | **MEAN** | **SD** | **MEAN** | **SD** |
| **C-che** Correct response for chest | 0,80 | 0,13 | 0,83 | 0,16 | 0,76 | 0,14 |
| **C-arm** Correct response for arm | 0,77 | 0,12 | 0,82 | 0,10 | 0,70 | 0,13 |
| **MSI** Multisensory Integration Task | | | | | | |
| **Variables** | **MEAN** | **SD** | **MEAN** | **SD** | **MEAN** | **SD** |
| **AUC-av** Area Under the Curve audio-visual | 0,21 | 0,19 | 0,17 | 0,16 | 0,20 | 0,20 |
| **AUC-at** Area Under the Curve audio-tactile | 0,21 | 0,17 | 0,12 | 0,17 | 0,23 | 0,24 |
| **AUC-vt** Area Under the Curve visuo-tactile | 0,23 | 0,14 | 0,24 | 0,26 | 0,17 | 0,13 |

**1 Interoceptive Bodily Dimensions**

**1.1 Interoceptive Accuracy**

Interoceptive accuracy refers to the accuracy in detecting internal bodily sensations (Garfinkel et al., 2015). In this study we employed two tasks to measure participants’ cardiac interoceptive accuracy: the Heartbeat Detection task, also known as the Tapping or Tracking task (Brener & Ring, 2016; McFarland RA., 1975), and the Heartbeat Counting task (R. Schandry, 1981). In the Heartbeat Detection task participants were seated comfortably in a chair with their feet flat on the ground. They were instructed to focus the attention on their heart and to press a button each time they perceive a heartbeat. The task consisted of two trials of two minutes each, with a short pause between them.

The Heartbeat Counting task measures cardiac interoceptive accuracy by evaluating participants' performance in silently counting their felt heartbeats during specific time intervals (Garfinkel et al., 2015). The task consists of four trials with different durations (25, 30, 45 and 100 seconds)(Pollatos et al., 2008). The trials were presented in a random order for each participant with a short pause between two consecutive trials. Each trial begins with a 10-second introductory phase to allow participants to settle into the task. After each trial, participants were asked to verbally report number of heartbeats they counted or estimated. The accuracy score (Acc-c) for each participant was obtained comparing the number of R-peaks recorded with ECG to the number of heartbeats reported during each trial (Koch et al., 2014).

**1.2 Interoceptive Sensibility**

Interoceptive sensibility is the subjective account of experiencing internal bodily sensations (Critchley & Garfinkel, 2017). It can be assessed using subjective measures that index both the individual's confidence in their interoceptive ability and their interoceptive feelings (Garfinkel et al., 2015). We assessed participants' interoceptive sensibility based on their confidence in interoceptive accuracy during the Heartbeat Detection task (Con-d) and the Heartbeat Counting task (Con-c). The confidence was assessed using a scale ranging from 1 to 9, with 1 indicating low confidence (total guess/no heartbeat awareness) and 9 indicating high confidence (complete perception of heartbeat).

To assess interoceptive sensibility we also used two self-report questionnaires: the Body Perception Questionnaire-22 (BPQ-22)(Poli et al., 2021; Porges, 1993) and the Multidimensional Assessment of Interoceptive Awareness (MAIA)(Calì et al., 2015; Mehling et al., 2012). The BPQ-22 is a 22-item scale that evaluates body awareness and autonomic symptoms across three sub-dimensions: the body awareness (BOA), related to the upper parts of the body or to the whole body, the supradiaphragmatic reactivity (SUP), involved in regulating the functions of organs situated above the diaphragm, and the subdiaphragmatic/body awareness factor (BOA/SUB), including items related to subdiaphragmatic issues. The MAIA is 32-item self-report questionnaires to assess multiple sub-dimensions of interoceptive sensibility. From the MAIA we obtained a score for each subscale: Noticing (1M), Not-distracting (2M), Not-worrying (3M), Attention regulation (4M), Emotional awareness (5M), Self-regulation (6M), Body listening (7M) and Trusting (8M).

**1.3 Interoceptive Awareness**

Interoceptive awareness is the metacognitive awareness of interoceptive accuracy and refers to the correspondence between objective measure of interoceptive accuracy and subjective confidence (Garfinkel et al., 2015; Suzuki et al., 2013). We assessed participant’s interoceptive awareness comparing the accuracy and the confidence in both the Heartbeat Detection task (Aw-d) and the Heartbeat Counting task (Aw-c).

**2 Exteroceptive Bodily Dimensions**

**2.1 Body Image**

Body image refers to the subjective experience of the physical structure of our body in terms of its size, shape, and physical composition (Longo, 2016). We assessed body image through the Photographic Figure Rating Scale (PFRS) task, adapted from Naor-Ziv et al.(Naor-Ziv et al., 2020). During this task, participants were shown ten silhouette images representing a wide range of body sizes, ranging from extremely thin to extremely obese. The ten images were calibrated with specific Body Mass Index (BMI) values, ranging from a BMI of 16 (image 1) to a BMI of 40 (image 10)(Pasciucco et al., 2025; Swami et al., 2008). This calibration provided a standardized representation of body sizes in the scale. Each participant was shown the series of ten images twice: once in ascending order (from thinnest to heaviest) and once in descending order (from heaviest to thinnest). Participants were instructed to select a single image based on their perceived similarity to their own current physique. We then calculated the participant's actual physique (A) as the mean of the BMIs represented in the two images selected in the task. Lastly, we calculated the difference between the real BMI (R) of each participant, based on their height and weight, and the actual physique (A), denoted as ΔAR (A-R).

**2.2 Spatial Tactile Acuity**

Spatial tactile acuity refers to the ability to precisely perceive the location and the quality of touch (Harvie et al., 2018). This dimension was evaluated through the Two-point discrimination (2PD) task, which assesses the ability of participants to identify two closely spaced points on a small area of the skin and the accuracy of their discrimination skills (Weber, 1996). We compared two body parts used as targets areas, the chest and the arm, with a third one, the neck, used as reference (Spitoni et al., 2015). The experiment consisted of 30 trials per condition, resulting in a total of trials 60 (20 trials per size). During each trial, the reference stimulus was a fixed distance of 5 cm. On the target body part, the distance between the contact points could vary between 4, 5, or 6 cm. The task assigned to the participants was to indicate which of the two stimuli they perceived as having a greater amplitude. From this task, we quantified the correct response for the two target body parts, chest (C-che) and arm (C-arm).

**2.3 Body Structural Representation**

Body structural representation refers to the knowledge about the topological organization of own bodies, outlining how different body parts interrelate within a spatial configuration, focusing on the spatial positioning of each body part related to others (Longo, 2016). This dimension was evaluated through the Finger Localization task (Benton, 1983), which requires participants to identify and differentiate which fingers were stimulated in three different conditions.

During the Finger Localization task, participants were seated in front of a table with their hands resting on the surface, palms facing upwards, and fingers extended and slightly separated. In the first part (a), participants were required to identify whether the experimenter touched a finger on their left hand or their right hand. In the second part (b), participants had to identify which finger of their left or right hand was touched by the experimenter, without being able to see their hands. In the third part (c), participants had to identify pairs of fingers on their left or right hand that were touched by the experimenter, without being able to see their hands. Each task included ten trials for each hand. For each trial, participants indicated their responses by pointing on an outline of a human hand, correct responses were recorded for each trial, and the maximum total score was 60 (Costantini et al., 2020; Fotia et al., 2022). In this study, we focused on the third part, calculating the accuracy as the number of correct responses (HIT) across all the trials, with a maximum score of 20.

**2.4 Multisensory Integration**

Multisensory integration refers to the process by which inputs from two or more sensory modalities are combined by the nervous system to form a stable and coherent percept of the world (Yau et al., 2015). This dimension was evaluated through the Multisensory Integration (MSI) task aiming at investigating the integration of visual, auditory, and tactile stimuli in participants' perception. Visual stimulation was achieved by incorporating a LED light placed inside a box. Tactile stimulation was provided using a constant-current electrical stimulator (Digitimer DS7A), delivering electrical pulses via two electrodes attached to the participant's right middle finger. The auditory stimulation was introduced by playing brief auditory beeps. The experiment consisted of six different conditions: visual-only (V), tactile-only (T), auditory-only (A), visuo-tactile (VT), audio-visual (AV), and audio-tactile (AT). Each condition included 30 trials. The conditions were presented in a randomized order. A foot pedal response pad was positioned under participants’ right foot. They were asked to press the pedal as quickly as possible upon perceiving any stimulus presented during the task (Mahoney et al., 2015). For each multisensory pair, audio-visual (AV), audio-tactile (AT) and visuo-tactile (VT), we calculated the area-under-the-curve (AUC) based on the distribution of reaction times to multisensory stimuli as compared to the distribution of reaction times to unimodal stimuli. Hence, AUC-av, AUC-at and AUC-vt represent a proxy of the magnitude of multisensory integration for audio-visual, audio-tactile and visuo-tactile stimuli, respectively (Mahoney et al., 2019).

**2.5 Multisensory Temporal Resolution**

Multisensory temporal resolution refers to the principle that optimal multisensory integration occurs when stimuli from different sensory modalities are presented closely in time. This principle highlights the critical role of temporal proximity in influencing the nervous system's integration of diverse sensory information (Sarko et al., 2012).

This dimension was evaluated through the Simultaneity Judgment (SJ) task, which aims to measure temporal sensitivity in the integration of multisensory stimuli. Specifically, we focused on auditory and tactile stimuli (AT). Auditory stimuli were delivered through headphones and each stimulus consisted of a 3,500-Hz pure tone lasting 30ms. Tactile stimuli were administered using two constant-current electrical stimulators (Digitimer DS7A), which controlled two pairs of neurological electrodes attached to the participant's right and left middle fingers. Tactile stimuli were single, constant voltage, rectangular monophasic pulses lasting 100 milliseconds (ms). During the task participants were blindfolded and seated in front of a table, with their index fingers resting on two separate keys of a response box. The intensity of the stimuli was adjusted to each participant's detection threshold, set at 100% for both auditory and tactile stimuli. In each trial, two stimuli were presented in opposite hemispaces, with stimulus pairs randomly interleaved at various stimulus onset asynchronies (SOAs): ±450, ±350, ±200, ±120, ±70, ±40, ±15, and 0 ms. Participants had to indicate whether the two stimuli were presented simultaneously or not using the response box. The AT-SJ task comprised two blocks of 120 trials each, resulting in a total of 240 trials, 16 trials per each SOA. The association between the hemispace of presentation (right or left) and the modality (auditory or tactile) was balanced across trials, and the stimulus-response button association was counterbalanced between blocks.

Based on participants responses, we calculated the following parameters: *a*) the point of subjective equality (PSE), providing an estimate of the interval between stimuli at which there is the highest probability of the perception of simultaneity and *b*) the just noticeable difference (JND-sj), reflecting the minimal temporal interval at which the change between the perceived temporal relation stimuli can be observed (Binder, 2015).

**2.6 Peripersonal Space**

Peripersonal space refers to the space surrounding one’s own body, where the integration of stimuli on the body and from the external environment is facilitated (Rabellino et al., 2020). We evaluated this dimension through the Peripersonal Space (PPS) task (Canzoneri et al., 2012; Di Cosmo et al., 2018, 2021), which measures individual peripersonal space boundaries by assessing the optimal temporal interval for the integration of tactile and auditory stimuli (Ferri et al., 2015; Spadone et al., 2021). During this task participants were blindfolded and seated comfortably at a table with their right hand resting on surface, palm facing downward. Participants wore headphones and a constant-current electrical stimulator (Digitimer DS7A) that controlled a pair of electrodes was attached to participants’ right middle finger. Each experimental trial involved the presentation of a looming sound along with a tactile stimulus. The tactile stimulus was delivered at five different time delays from the onset of the auditory stimulus (300, 800, 1500, 2200 and 2700 ms). Tactile stimulation could also be delivered during the silent periods preceding the presentation of the sound (-700 ms). The remaining trials were catch trials, involving only auditory stimulation. This task consisted of 80 trials, including 50 post-trials (10 for each delay), 10 pre-trials, and 20 catch trials. Participants were instructed to respond as quickly as possible to the tactile stimulus pressing a button on a response box with their left index finger. They were required to ignore the auditory stimulus. After each trial, there was an intertrial interval of 1000 ms, allowing to rest and prepare for the next trial (Ardizzi & Ferri, 2018; Canzoneri et al., 2012; Di Cosmo et al., 2021). To estimate individual peripersonal space boundary, a psychometric function is fitted to the reaction time (RT) data (Miller, J. & Ulrich, L., 2001). The PSE of the psychometric function serves as a proxy of the peripersonal space boundary.

**2.7 Temporal Tactile Acuity**

Temporal tactile acuity is the ability to detect and distinguish temporal characteristics of sensations related to touch (Laasonen et al., 2001). We assessed this dimension through the Temporal Order Judgment (TOJ) task. In the TOJ task, participants have to determine the order of two tactile stimuli presented sequentially to their hands. When the hands are in an “uncrossed” position (toju), participants can rely on tactile and proprioceptive cues related to their body posture, utilizing a body-centred reference frame(Heed et al., 2014). During TOJ, participants were blindfolded and seated at a table with their hands placed on the surface, palm down. Two tactile stimuli, one for each hand, were presented in rapid succession. In each trial, the SOA between the two stimuli was randomly assigned from 22 intervals ranging from -450 ms to 450 ms (±450, ±300, ±200, ±150, ±100, ±75, ±50, ±40, ±30, ±15, ±5 ms). Negative and positive intervals indicated whether the left or right hand was stimulated first. The inter-trial intervals varied between 2000 and 4000 ms to ensure that each trial has the same duration. The task comprised 176 trials, with eight trials per SOA(Ferri et al., 2016). Tactile stimuli were delivered using two constant-current electrical stimulators (Digitimer DS7A), which controlled pairs of neurological electrodes attached to the dorsal surface of the middle fingers. The stimuli were supra-threshold vibrotactile stimuli oscillating at 100 Hz, with a total duration of 30 ms. They were instructed to make two-alternative forced-choice judgments regarding the order of stimulation by pressing the button under the index finger of the hand they believed was stimulated earlier or later than the other. The two response strategies (earlier and later) were counterbalanced. Trials with response times exceeding 3000 ms or without a response were re-presented at the end of the corresponding block, ensuring an equal number of trials across all the participants. Participants did not receive feedback regards their performance. From this task, we quantified the JND (JND-toju), as a measure of precision, and the proportion of correct responses (Azañón et al., 2016). The JND-toju represents the smallest interval at which the participants can reliably decide which sensory input of the two presented was first (Kostaki M. & Vatakis A., 2018).

**2.8 Sensorimotor functions**

*Touch remapping*

To perceive the location of touch in space, the brain combines information about touched skin location with information about the location of that body part in space. When the two hands are in a “crossed” position, this integration is impaired, affecting the ability to judge the order of touches on both hands (Azañón et al., 2016), creating a conflict between how tactile senses represent external space. Consequently, the same cues must be remapped using an external reference frame. This remapping process becomes necessary to accurately judge the temporal order of the tactile stimuli in the crossed-hand condition (tojc)(Ferri et al., 2016). Participants performed the TOJ task under a crossed-hand condition, crossing their arms over their wrists. The two possible arm positions (right over left and vice versa) were counterbalanced across participants, and the order was randomly assigned to each participant. Two tactile stimuli were presented in rapid succession, with one stimulus delivered to each hand. The time interval between the two stimuli ranged from -900 to 900 ms (±900, ±600, ±400, ±300, ±200, ±150, ±100, ±80, ±60, ±30, ±10 ms). From this task, we measured the following variables: the JND (JND-tojc), which represents the smallest interval at which participants can reliably determine which of the two presented sensory inputs came first (Kostaki M. & Vatakis A., 2018) and the Sum of Confusions (SC-toj), that indicates the sum of differences in the response functions between crossed and uncrossed conditions (Ferri et al., 2016). SC-toj is a global indicator of differences, which provides an overarching measure of the divergence between the two conditions and assess increases in judgment reversals resulting from the arm-crossing manipulation (Wada et al., 2014).

*Laterality Judgment Task*

The Laterality Judgement Task (LJT) is designed to assess participants' ability to mentally rotate hand images and accurately judge the lateral orientation of presented hand images (Mibu et al., 2020). In this task, grey-scale pictures depicting the dorsal view of both right and left hands are presented individually on a screen. The hand images are displayed in six different clockwise orientations: upright 0°, 60°, 120°, 180°, 240°, and 300°, with the upright orientation representing the hand with the fingers pointing upwards. Participants seated in front of a screen at distance of approximately 40 cm. Each trial started with the presentation of a fixation cross for 500 ms to ensure participants' attention is focused. Following the fixation cross, a hand image was presented at the centre of the screen. The hand images represented either a left or a right hand. During the task, participants were instructed to judge the laterality (left or right) of the observed hand image as accurately and quickly as possible. They had a maximum response time window of 4000 ms. Participants used their left and right index fingers to press designated response keys that correspond to their judgment of the hand's laterality. The left key is pressed for a judgment of “left hand”, while the right key is pressed for a judgment of “right hand”(Ferri et al., 2011). The LJT consisted of 144 trials, with each orientation condition being presented 12 times (6 times for each hand). We calculated a slope, which reflect the efficiency of the neural mechanism underlying the mental rotation process: a smaller slope indicates higher neural efficiency in mental rotation (Christova et al., 2008). We quantified slopes for both hands, indicated as Mental Rotation Efficiency for the left hand (MRE-lh) and the right hand (MRE-rh), illustrating the efficiency of the respective mental rotation processes (Ferri et al., 2011).

**References**

Ardizzi, M., & Ferri, F. (2018). Interoceptive influences on peripersonal space boundary. *Cognition*, *177*, 79–86. https://doi.org/10.1016/J.COGNITION.2018.04.001

Azañón, E., Mihaljevic, K., & Longo, M. R. (2016). A three-dimensional spatial characterization of the crossed-hands deficit. *Cognition*, *157*, 289–295. https://doi.org/10.1016/J.COGNITION.2016.09.007

Bausenhart, K. M., Luca, M. Di, & Ulrich, R. (n.d.). *Assessing Duration Discrimination: Psychophysical Methods and Psychometric Function Analysis*.

Benton, A. L. , H. K. , V. N. R. , S. O. ,. (1983). *Contributions to neuropsychological assessment: tests: 9. Finger localization complete test.* (Oxford University Press, Ed.).

Binder, M. (2015). Neural correlates of audiovisual temporal processing--comparison of temporal order and simultaneity judgments. *Neuroscience*, *300*, 432–447. https://doi.org/10.1016/J.NEUROSCIENCE.2015.05.011

Brener, J., & Ring, C. (2016). Towards a psychophysics of interoceptive processes: The measurement of heartbeat detection. In *Philosophical Transactions of the Royal Society B: Biological Sciences* (Vol. 371, Issue 1708). Royal Society of London. https://doi.org/10.1098/rstb.2016.0015

Calì, G., Ambrosini, E., Picconi, L., Mehling, W. E., & Committeri, G. (2015). Investigating the relationship between interoceptive accuracy,interoceptive awareness,and emotional susceptibility. *Frontiers in Psychology*, *6*. https://doi.org/10.3389/fpsyg.2015.01202

Canzoneri, E., Magosso, E., & Serino, A. (2012). Dynamic Sounds Capture the Boundaries of Peripersonal Space Representation in Humans. *PLOS ONE*, *7*(9), e44306. https://doi.org/10.1371/JOURNAL.PONE.0044306

Christova, P. S., Lewis, S. M., Tagaris, G. A., Uğurbil, K., & Georgopoulos, A. P. (2008). A voxel-by-voxel parametric fMRI study of motor mental rotation: Hemispheric specialization and gender differences in neural processing efficiency. *Experimental Brain Research*, *189*(1), 79–90. https://doi.org/10.1007/S00221-008-1405-X

Costantini, M., Salone, A., Martinotti, G., Fiori, F., Fotia, F., Di Giannantonio, M., & Ferri, F. (2020). Body representations and basic symptoms in schizophrenia. *Schizophrenia Research*, *222*, 267–273. https://doi.org/10.1016/j.schres.2020.05.038

Critchley, H. D., & Garfinkel, S. N. (2017). Interoception and emotion. *Current Opinion in Psychology*, *17*, 7–14. https://doi.org/10.1016/J.COPSYC.2017.04.020

Di Cosmo, G., Costantini, M., Salone, A., Martinotti, G., Di Iorio, G., Di Giannantonio, M., & Ferri, F. (2018). Peripersonal space boundary in schizotypy and schizophrenia. In *Schizophrenia Research* (Vol. 197, pp. 589–590). Elsevier B.V. https://doi.org/10.1016/j.schres.2017.12.003

Di Cosmo, G., Costantini, M., Spadone, S., Pizzella, V., Della Penna, S., Marzetti, L., & Ferri, F. (2021). Phase-coupling of neural oscillations contributes to individual differences in peripersonal space. *Neuropsychologia*, *156*. https://doi.org/10.1016/j.neuropsychologia.2021.107823

Ferri, F., Ambrosini, E., & Costantini, M. (2016). Spatiotemporal processing of somatosensory stimuli in schizotypy. *Scientific Reports*, *6*. https://doi.org/10.1038/srep38735

Ferri, F., Costantini, M., Huang, Z., Perrucci, M. G., Ferretti, A., Romani, G. L., & Northoff, G. (2015). Intertrial variability in the premotor cortex accounts for individual differences in peripersonal space. *Journal of Neuroscience*, *35*(50), 16328–16339. https://doi.org/10.1523/JNEUROSCI.1696-15.2015

Ferri, F., Frassinetti, F., Costantini, M., & Gallese, V. (2011). Motor Simulation and the Bodily Self. *PLoS ONE*, *6*(3), e17927. https://doi.org/10.1371/journal.pone.0017927

Fotia, F., Van Dam, L., Sykes, J. J., Ambrosini, E., Costantini, M., & Ferri, F. (2022). Body structural representation in schizotypy. *Schizophrenia Research*, *239*, 1–10. https://doi.org/10.1016/j.schres.2021.11.002

Garfinkel, S. N., Seth, A. K., Barrett, A. B., Suzuki, K., & Critchley, H. D. (2015). Knowing your own heart: Distinguishing interoceptive accuracy from interoceptive awareness. *Biological Psychology*, *104*, 65–74. https://doi.org/10.1016/J.BIOPSYCHO.2014.11.004

Harvie, D. S., Edmond-Hank, G., & Smith, A. D. (2018). Tactile acuity is reduced in people with chronic neck pain. *Musculoskeletal Science and Practice*, *33*, 61–66. https://doi.org/10.1016/j.msksp.2017.11.009

Heed, T., Azañón, E., Jones, A., & Ricciardi, E. (2014). *Using time to investigate space: a review of tactile temporal order judgments as a window onto spatial processing in touch*. https://doi.org/10.3389/fpsyg.2014.00076

Koch, A., Pollatos, O., Mehling, W. E., Sänger, J., & Dunn, B. (2014). *Interoceptive sensitivity, body weight and eating behavior in children: a prospective study*. https://doi.org/10.3389/fpsyg.2014.01003

Kostaki M., V. A., & Vatakis A., B. F. , D. L. M. , C. Á. (2018). Temporal order and synchrony judgments: a primer for students, in Timing and Time Perception: Procedures, Measures, and Applications. *(Leiden: BRILL Press;*, 233–262.

Laasonen, M., Service, E., & Virsu, V. (2001). Temporal order and processing acuity of visual, auditory, and tactile perception in developmentally dyslexic young adults. *Cognitive, Affective & Behavioral Neuroscience*, *1*(4), 394–410. https://doi.org/10.3758/CABN.1.4.394

Longo, M. R. (2016). *Types of body representation* (In Y. Coello & M. H. Fischer (Eds.), Ed.; pp. 117–134).

Mahoney, J. R., Cotton, K., & Verghese, J. (2019). Medical Sciences cite as. *J Gerontol A Biol Sci Med Sci*, *74*(9), 1429–1435. https://doi.org/10.1093/gerona/gly245

Mahoney, J. R., Dumas, K., & Holtzer, R. (2015). *Visual-Somatosensory Integration is Linked to Physical Activity Level in Older Adults*.

McFarland RA. (1975). Heart rate perception and heart rate control. *Psychophysiology* , *12*, 402–405. https://doi.org/10.1111/j.1469-8986.1975.tb00011.x

Mehling, W. E., Price, C., Daubenmier, J. J., Acree, M., & Bartmess, E. (2012). The Multidimensional Assessment of Interoceptive Awareness (MAIA). *PLoS ONE*, *7*(11), 48230. https://doi.org/10.1371/journal.pone.0048230

Mibu, A., Kan, S., Nishigami, T., Fujino, Y., & Shibata, M. (2020). Performing the hand laterality judgement task does not necessarily require motor imagery. *Scientific Reports*, *10*(1). https://doi.org/10.1038/s41598-020-61937-9

Miller, J., & Ulrich, R. (2001). On the analysis of psychometric functions: The Spearman-Kärber method. Perception & Psychophysics, 63(8), 1399–1420. <https://doi.org/10.3758/BF03194551>

Naor-Ziv, R., King, R., & Glicksohn, J. (2020). Rank-order of body shapes reveals internal hierarchy of body image. *Journal for Person-Oriented Research*, *6*(1), 28–38. https://doi.org/10.17505/jpor.2020.22044

Pasciucco, M. R., Perrucci, M. G., Croce, P., Kalckert, A., Costantini, M., & Ferri, F. (2025). Predictive role of exteroceptive and interoceptive bodily dimensions to schizotypal personality traits. *Scientific Reports*, *15*(1), 7909. https://doi.org/10.1038/S41598-025-89951-9

Poli, A., Maremmani, A. G. I., Chiorri, C., Mazzoni, G. P., Orrù, G., Kolacz, J., Porges, S. W., Conversano, C., Gemignani, A., & Miccoli, M. (2021). Item reduction, psychometric and biometric properties of the italian version of the body perception questionnaire—short form (Bpq-sf): The bpq-22. *International Journal of Environmental Research and Public Health*, *18*(7). https://doi.org/10.3390/ijerph18073835

Pollatos, O., Kurz, A. L., Albrecht, J., Schreder, T., Kleemann, A. M., Schöpf, V., Kopietz, R., Wiesmann, M., & Schandry, R. (2008). Reduced perception of bodily signals in anorexia nervosa. *Eating Behaviors*, *9*(4), 381–388. https://doi.org/10.1016/j.eatbeh.2008.02.001

Porges, S. (1993). Body perception questionnaire. . *Laboratory of Developmental Assessment, University of Maryland, 10,* .

R. Schandry. (1981). Heart beat perception and emotional experience. *Psychophysiology, 18 (4)* , 483–488.

Rabellino, D., Frewen, P. A., McKinnon, M. C., & Lanius, R. A. (2020). Peripersonal Space and Bodily Self-Consciousness: Implications for Psychological Trauma-Related Disorders. In *Frontiers in Neuroscience* (Vol. 14). Frontiers Media S.A. https://doi.org/10.3389/fnins.2020.586605

Sarko, D. K., Nidiffer, A. R., Powers, A. R., Ghose, D., Hillock-Dunn, A., Fister, M. C., Krueger, J., & Wallace, M. T. (2012). Spatial and Temporal Features of Multisensory Processes. *The Neural Bases of Multisensory Processes*, 191–215. https://www.ncbi.nlm.nih.gov/books/NBK92831/

Spadone, S., Perrucci, M. G., Di Cosmo, G., Costantini, M., Della Penna, S., & Ferri, F. (2021). Frontal and parietal background connectivity and their dynamic changes account for individual differences in the multisensory representation of peripersonal space. *Scientific Reports*, *11*(1), 20533. https://doi.org/10.1038/s41598-021-00048-5

Spitoni, G. F., Serino, A., Cotugno, A., Mancini, F., Antonucci, G., & Pizzamiglio, L. (2015). The two dimensions of the body representation in women suffering from Anorexia Nervosa. *Psychiatry Research*, *230*(2), 181–188. https://doi.org/10.1016/j.psychres.2015.08.036

Suzuki, K., Garfinkel, S. N., Critchley, H. D., & Seth, A. K. (2013). Multisensory integration across exteroceptive and interoceptive domains modulates self-experience in the rubber-hand illusion. *Neuropsychologia*, *51*(13), 2909–2917. https://doi.org/10.1016/j.neuropsychologia.2013.08.014

Swami, V., Salem, N., Furnham, A., & Tovée, M. J. (2008). Initial examination of the validity and reliability of the female photographic figure rating scale for body image assessment. *Personality and Individual Differences*, *44*(8), 1752–1761. https://doi.org/10.1016/j.paid.2008.02.002

Wada, M., Suzuki, M., Takaki, A., Miyao, M., Spence, C., & Kansaku, K. (2014). *Spatio-temporal processing of tactile stimuli in autistic children*. https://doi.org/10.1038/srep05985

Weber, E. H. (1996). *ON THE TACTILE SENSES*. https://doi.org/https://doi.org/10.4324/9781315782089

Yau, J. M., DeAngelis, G. C., & Angelaki, D. E. (2015). Dissecting neural circuits for multisensory integration and crossmodal processing. *Philosophical Transactions of the Royal Society B: Biological Sciences*, *370*(1677). https://doi.org/10.1098/RSTB.2014.0203
